# Pressure-induced transformation of CH_3_NH_3_PbI_3_: the role of the noble-gas pressure transmitting media

**DOI:** 10.1107/S2052520619004554

**Published:** 2019-05-18

**Authors:** Alla Arakcheeva, Volodymyr Svitlyk, Eleonora Polini, Laura Henry, Dmitry Chernyshov, Andrzej Sienkiewicz, Gaétan Giriat, Anastasiia Glushkova, Marton Kollar, Bálint Náfrádi, Laszlo Forro, Endre Horváth

**Affiliations:** a Ecole Polytechnique Fédérale de Lausanne, School of Basic Sciences, Institute of Physics, Laboratory of Physics of Complex Matter (SB IPHYS LPMC), PH D2 445 (Bâtiment PH), Station 3, Lausanne, CH-1015, Switzerland; b ID27 High Pressure Beamline, ESRF, 71 Avenue des Martyrs, Cedex 9, Grenoble, 38043, France; c Department of Physics, Università di Roma La Sapienza, Piazzale Aldo Moro, 5, Roma RM, 00185 Italy; d SNBL, ESRF, 71 Avenue des Martyrs, Cedex 9, Grenoble, 38043, France; e ADSresonances SARL, Route de Genève 60B, Préverenges, CH-1028, Switzerland

**Keywords:** methyl­ammonium lead iodide, crystal structure, high pressure, Ne and Ar pressure transmitting media

## Abstract

The photovoltaic perovskite, methyl­ammonium lead triiodide [CH_3_NH_3_PbI_3_ (MAPbI_3_)], is one of the most efficient materials for solar energy conversion. Various kinds of chemical and physical modifications have been applied to MAPbI_3_ towards better understanding of the relation between composition, structure, electronic properties and energy conversion efficiency of this material. Pressure is a particularly useful tool, as it can substantially reduce the interatomic spacing in this relatively soft material and cause significant modifications to the electronic structure. Application of high pressure induces changes in the crystal symmetry up to a threshold level above which it leads to amorphization. Here, a detailed structural study of MAPbI_3_ at high hydro­static pressures using Ne and Ar as pressure transmitting media is reported. Single-crystal X-ray diffraction experiments with synchrotron radiation at room temperature in the 0–20 GPa pressure range show that atoms of both gaseous media, Ne and Ar, are gradually incorporated into MAPbI_3_, thus leading to marked structural changes of the material. Specifically, Ne stabilizes the high-pressure phase of Ne_*x*_MAPbI_3_ and prevents amorphization up to 20 GPa. After releasing the pressure, the crystal has the composition of Ne_0.97_MAPbI_3_, which remains stable under ambient conditions. In contrast, above 2.4 GPa, Ar accelerates an irreversible amorphization. The distinct impacts of Ne and Ar are attributed to differences in their chemical reactivity under pressure inside the restricted space between the PbI_6_ octahedra.

## Abstract 2

A structural study of methyl­ammonium lead triiodide [CH_3_NH_3_PbI_3_ (MAPbI_3_)], at high pressures up to 20 GPa using noble gases Ne and Ar as pressure-transmitting media is reported. It is found that both noble gases are chemically active at high pressures. In particular, Ne stabilizes the high-pressure structure of Ne_*x*_MAPbI_3_ and prevents amorphization up to 20 GPa. In contrast, Ar acts as a stabilizer only up to 2.4 GPa and accelerates irreversible amorphization upon further compression.

## 1. Introduction   

**Figure 1:**
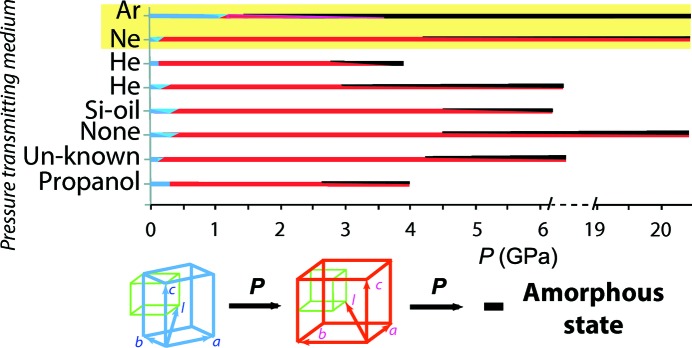
Schematic overview of the pressure-induced transformation of MAPbI_3_ at room temperature. Top panel: influence of the PTM on the transformation. The thickness of the red and black lines represents schematically the relative content of the (pseudo) cubic and amorphous phases shown, respectively, in the bottom panel. Bottom panel: relations between the unit cells of different phases. Unit-cell parameters: *a*_pr_ ≃ 6.4 Å for the primitive perovskite cubic cell (green line); *a* = *b* ≃ *a*_pr_ 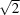 ≃ 8.8 Å and *c* ≃ 2*a*_pr_ ≃ 12.7 Å for the tetragonal body-centred cell (blue) under ambient condition; *a* ≃ *b* ≃ *c* ≃ 2*a*_pr_ ≃ 12.3 Å for the body-centred (pseudo)cubic cell (red) under high pressure. The data are taken from: Capitani *et al.* (2016 ▸) and Jaffe *et al.* (2016 ▸) for He; Ou *et al.* (2016 ▸) and Jiang *et al.* (2016 ▸) for no PTM (None); Jiang *et al.* (2016 ▸) for silicon oil (Si-oil); Wang *et al.* (2015 ▸) for non-specified PTM (Unknown); Szafrański & Katrusiak (2016 ▸) for propanol. Data corresponding to Ar and Ne (yellow background) are from the present work.

MAPbI_3_ is currently considered as one of the most promising compounds in photovoltaic technologies for making cheap and highly efficient solar cells (Green *et al.*, 2014 ▸; Weber *et al.*, 2015 ▸). One of the hurdles which reduces the enthusiasm for practical applications of MAPbI_3_ is its content of lead which confers toxicity on this material (Benmessaoud *et al.*, 2016 ▸). Therefore, it would be highly desirable to replace lead with a non-toxic element, while still preserving the high light conversion efficiency. The *conditio sine qua non* for achieving this goal is a better understanding of the microscopic origin of this high energy conversion efficiency. There are opinions that the rotation or other type of motion of methyl­ammonium cations (MA) introduces a slightly indirect band gap in this material, which extends the lifetime of photoelectrons (Motta *et al.*, 2015 ▸), helping them to flow out of the material as a current. Valuable information about the role of the cation can be obtained by studying its behaviour in the lattice under high pressure (Ou *et al.*, 2016 ▸). The perovskite crystal structure of MAPbI_3_ is very flexible due to a large interstitial space (of ∼8 Å in diameter), where the small (∼1.5 Å) linear cation is located. The PbI_6_ octahedra and the cations interact *via* weak hydrogen bonds. The strength and the conformation of the hydrogen bonds can be influenced either by the presence of captured exogenous substances, such as water (Arakcheeva *et al.*, 2016 ▸), or by external factors such as pressure or temperature, leading to changes in the symmetry of the unit cell. A recently published extended review (Lü *et al.*, 2017 ▸) on the pressure-induced evolution of the structure and the physical properties of organic–inorganic halide perovskites demonstrates a poor reproducibility of the changes. This calls for more investigations to uncover the underlying mechanisms. Fig. 1 ▸ shows a schematic representation of the two major pressure-induced structural transformations in MAPbI_3_, a phase transition and amorphization, reported in the literature together with the ones obtained in the present study. At very low pressures (0.1–0.3 GPa), a first-order phase transition from the tetragonal body-centred to the body-centred (pseudo) cubic unit cell takes place (Fig. 1 ▸, blue to red lines). The amorphization of the compound occurs after the structural phase transition (Fig. 1, black line). However, in different experiments the onset of the amorphization has been observed under different pressures, and the pressure transmitting medium (PTM) could be considered as a factor affecting the pressure-induced transformation of MAPbI_3_ (Fig. 1 ▸). Such an effect has already been observed in organic compounds (Zakharov *et al.*, 2016 ▸) and also in some minerals (Ardit *et al.*, 2014 ▸; Sato *et al.*, 2013 ▸; Guńka *et al.*, 2015 ▸; Lobban *et al.*, 2002 ▸). This provided the motivation for the present work on studying the influence of PTM on the structure of MAPbI_3_. We have chosen two noble gaseous media, Ne and Ar, which are among the most hydro­static PTMs and have distinct atomic radii: Ne (0.38 Å) and Ar (0.71 Å). Single-crystal synchrotron X-ray diffraction (XRD) measurements provided a precise data set, which allowed structure determination as a function of pressure with the best precision. Surprisingly, we found that under high pressure both Ar and Ne were incorporated into the structure. Moreover, the pressure-induced phase of Ne_0.97_MAPbI_3_ remained stable and highly crystalline after decompression.

## 2. Experimental   

### 2.1. Synthesis and crystallization   

The preparation of MAPbI_3_ single crystals has been reported elsewhere (Arakcheeva *et al.*, 2016 ▸). Several crystals were initially tested for their crystal structure and crystal quality at ambient conditions. All of them showed the space group *I*422 crystal symmetry with the unit-cell parameters and the atomic coordinates identical to those reported by Arakcheeva *et al.* (2016 ▸). Preliminary diffraction measurements under high pressure showed as expected that the starting quality of the crystal (degree of mosaic and strains) has a significant effect on the high-pressure transformation (Fig. S1). Therefore, only the highest quality crystals have been selected for the final diffraction experiments.

### 2.2. Single-crystal synchrotron XRD experiments   

**Figure 2:**
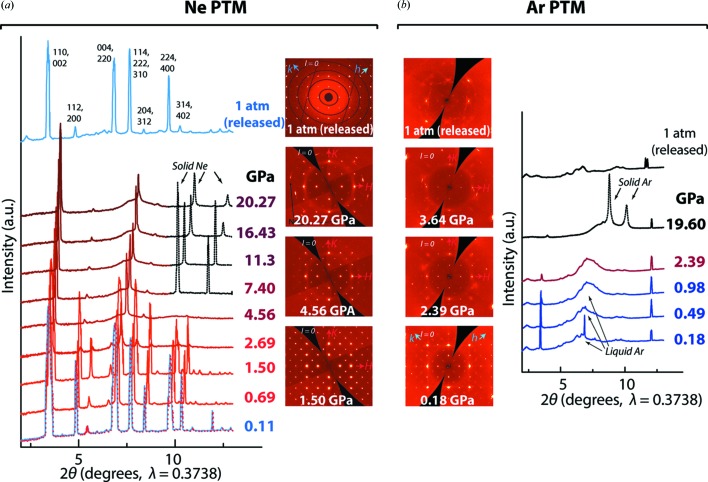
The XRD patterns illustrating the pressure-induced transformation of MAPbI_3_ with (*a*) Ne and (*b*) Ar as PTM. The powder XRD patterns calculated from the single-crystal experiments are presented at the logarithmic scale. Sections of the reciprocal space are shown for *l* = 0. The axes *h*, *k* (*H*, *K*) specific only for the tetragonal (pseudo-cubic) modification are emphasized in blue (red).

Room-temperature (293 K) high-pressure (up to 20.27 GPa) XRD data were collected at the ID27 High Pressure Beamline of the European Synchrotron Radiation Facility (ESRF) in Grenoble. Diamond anvil cells were used with rhenium gaskets and Ne or Ar gases as the PTM to generate hydro­static conditions. The X-ray wavelength was set to 0.3738 Å. The minimal linear size of the crystals was about 10 µm. Diffraction data were recorded with a Mar165 CCD detector and the pressure was measured using the ruby fluorescence technique (Syassen, 2008 ▸). After decompression, additional measurements were conducted on the Ne-containing crystal to determine its structure under ambient conditions. *CrysAlis PRO* (Rigaku Oxford Diffraction, 2014 ▸) and *JANA2006* (Petříček *et al.*, 2014 ▸) software packages were used for data processing and structural refinement, respectively. Experimental details are listed in Tables S1 and S2 and are illustrated in Fig. 2 ▸ and also in Figs. S1, S2 and S3.

#### 2.2.1. Structure determination   

**Figure 3:**
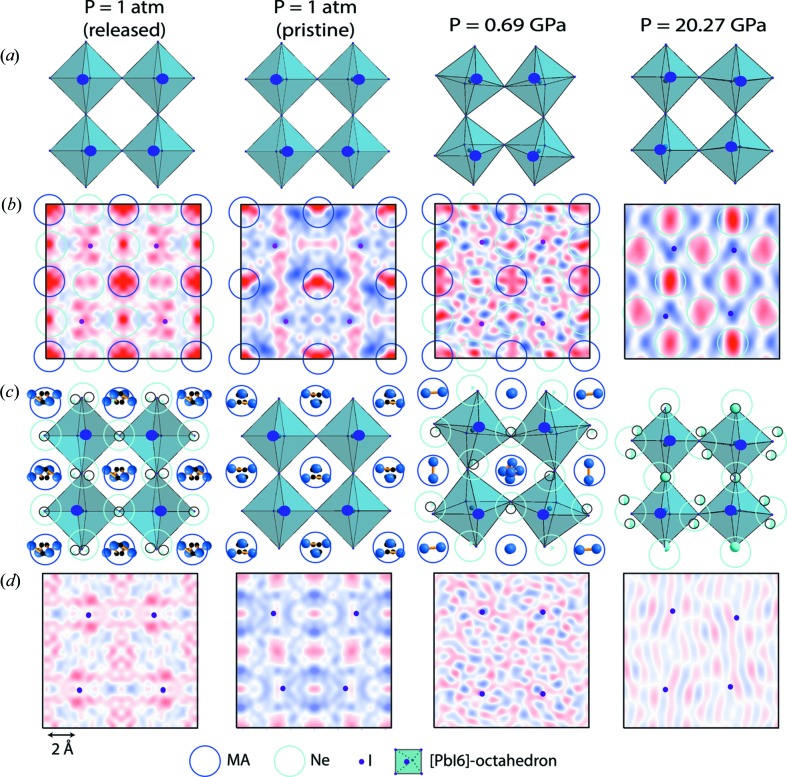
Determination of the MA and Ne positions in the crystal structure of MAPbI_3_ at selected pressures. Projections of the relevant fragments of the structure onto the *ac* plane are in (*a*) and (*c*). Small dots indicate I atoms in the plane for which the difference electron density maps are shown in (*b*) and (*d*) with positive (red) and negative (blue) areas. (*b*) The maps were calculated using only Pb and I atoms. The maxima of the difference electron densities are identified with MA cations (dark-blue circles) and Ne atoms (cyan circles). The density varies: from −0.32 to 1.58 e Å^−3^ for the released crystal at *P* = 1 atm; from 0.20 to 0.41 e Å^−3^ for the pristine crystal at *P* = 1 atm; from −1.44 to 1.97 e Å^−3^ for *P* = 0.69 GPa; from −1.13 to 1.86 e Å^−3^ for *P* = 20.27 GPa. (*d*) The residual electron density maps were calculated using all atoms of the structure. The residual electron density varies: from −0.3 to 0.51 e Å^−3^ atm for the released crystal at *P* = 1; from −0.27 to 0.23 e Å^−3^ for the pristine crystal at *P* = 1 atm; from −1.06 to 1.07 e Å^−3^ for *P* = 0.69 GPa; from −0.54 to 0.56 e Å^−3^ for *P* = 20.27 GPa.

The general scheme of the structure determination is illustrated in Fig. 3 ▸ using the example of the crystal at ambient conditions and under 0.69 GPa up to 20.27 GPa pressures with Ne as the PTM. The starting (pristine) and the released (after exposure to the highest pressure) crystals are shown next to each other for comparison. The most probable symmetry of the structure at each pressure was determined by testing the inorganic framework for all possible space groups in all possible crystallographic systems (cubic, tetragonal, trigonal and ortho­rhombic). Next, after the refinement of the atomic positions of Pb and I [Fig. 3 ▸(*a*)], difference electron density maps were calculated for each case [Fig. 3 ▸(*b*)]. The pristine crystal can be characterized by the maxima in the expected MA positions [dark-blue circles in Fig. 3 ▸(*b*)]. These maxima are also perfectly identified at 0.69 GPa and for the released crystal, whereas they are absent at 20.27 GPa. However, even stronger and more localized maxima [cyan circles in Fig. 3 ▸(*b*)] are clearly visible for each pressure, with the exception of the starting (pristine) case. These additional maxima are attributed to Ne atoms, giving a compatible electron density contribution [Fig. 3 ▸(*c*)]. At 20.27 GPa, the usual MA positions show nearly zero electron density and only the Ne-attributed maxima are observable. We have to emphasize that these maxima could not be fitted with displaced MA cations. The schematic diagrams of the crystal structure for each pressure after including MA and/or Ne in the refined models are shown in Fig. 3 ▸(*c*). The residual electron density maps calculated after the refinements have values close to zero [Fig. 3 ▸(*d*)], this supports the models.

Refinement of all atomic parameters for MA and Ne helped to confirm or correct the space group selected previously for the framework at each pressure. A similar procedure for the structure determination was used for all applied pressures for both Ne and Ar as the PTM.

As expected, the inclusion of Ne and Ar in the structure has a varying effect on the symmetry and the bulk modulus at different pressures. The resulting structures made it possible to determine the interatomic distances, and consequently the nature and hierarchy of interactions between various components of each structure.

**Figure 4:**
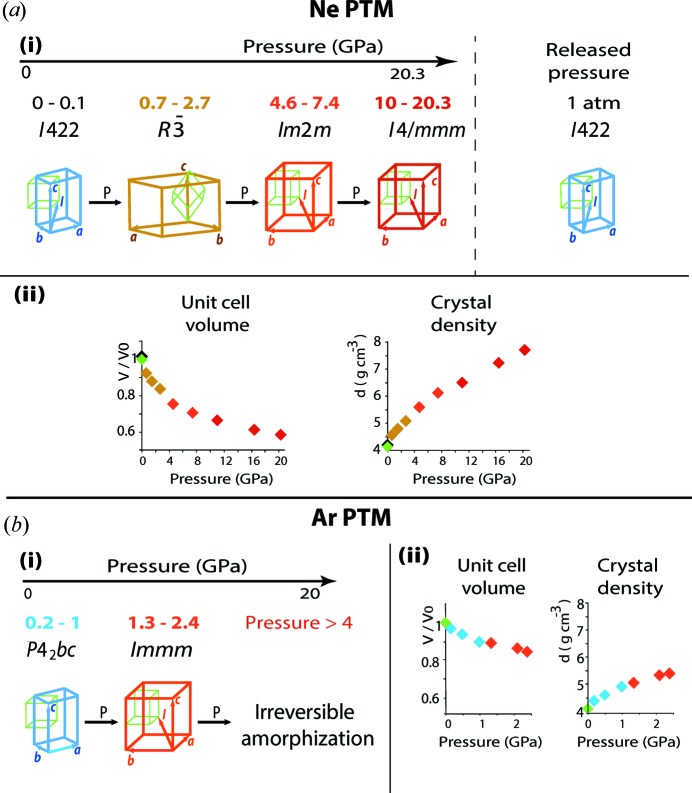
Evolution of crystallographic characteristics of MAPbI_3_ under pressure with (*a*) Ne and (*b*) Ar as the pressure transmitting media (PTM). Panels *a*(i) and *b*(i) show the symmetry transformation of the unit cell. Unit cells are shown in different colours for different crystallographic systems in relation to a primitive perovskite cube (green line). Panels *a*(ii), *b*(ii) show the pressure dependence of the unit-cell volume and crystal density. Points in the diagrams are coloured according to the unit cells in panels (i). Green and black points correspond to the pristine and released crystals, respectively.

The basic crystallographic information for eight different pressures with Ne as the PTM and six different pressures with Ar as the PTM is provided in Tables 1 ▸ and 2 ▸, respectively, and is visualized in Fig. 4 ▸. Further details are listed in Tables S1 and S2. It should be noted that, despite the reflections-to-parameters ratio which is 5.6 and 4.1 for the Ne-containing crystal at 16.43 and 20.27 GPa, respectively, the criteria *R*, *wR*, *S* = 0.051, 0.057, 1.44 for 16.43 GPa and 0.043, 0.064, 1.49 for 20.27 GPa, and the residual electron density ρ_max_, ρ_min_ = 0.58, −0.60 e Å^−3^ for 16.43 GPa and 1.06, −1.25 e Å^−3^ for 20.27 GPa (column VI and VII in Table S1) show that the corresponding structure solutions are correct. These structures obtained at the highest pressures are essential for understanding the Ne intercalation, which is also confirmed by the presence of Ne in the released crystal under ambient conditions.

## 3. Results and discussion   

### 3.1. The high-pressure NeMAPbI_3_ compounds   

The crystal structure at room temperature (293 K) was determined for 0.69, 1.5, 2.69, 4.56, 7.4, 16.43 and 20.27 GPa pressures and for ambient pressure after decompression (Table 1 ▸). Above 4 GPa an amorphous phase appears, coexisting with the crystalline one. The amorphous contribution slowly grows upon further increase of pressure and completely disappears after pressure is released (Fig. S1).

The XRD data obtained at 0.11 GPa indicate the coexistence of two phases: a tetragonal phase, characteristic of ambient pressure, and a pseudo-cubic phase, which is typical for higher pressures (Fig. S3). This is consistent with the first-order phase transition, which was reported before between 0.3 GPa and 0.4 GPa (Capitani *et al.*, 2016 ▸; Szafrański & Katrusiak, 2016 ▸; Francisco-López *et al.*, 2018 ▸). However, for the pseudo-cubic phase, we found the space group 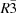, which differs from the cubic 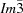 and orthorhombic *Imm*2 reported previously by Szafrański & Katrusiak (2016 ▸) and Capitani *et al.* (2016 ▸), respectively. We observed a second phase transformation between 2.69 GPa and 4.56 GPa. It is also in good agreement with the literature value of 2.5 GPa (Szafrański & Katrusiak, 2016 ▸) and 2.7 GPa (Francisco-López *et al.*, 2018 ▸). Even so, in contrast to the reported space group 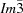 (Szafrański & Katrusiak, 2016 ▸), only its orthorhombic subgroup, *Im*2*m,* was found to describe the symmetry of the phase above 4.56 GPa. Furthermore, for the first time we observed a third transformation, from the orthorhombic to a tetragonal phase (space group *I*4/*mmm*) between 7.4 GPa and 16.43 GPa. The unit cells and space groups corresponding to each phase are shown in Fig. 4 ▸(*a*)(i). We explain the difference in the symmetry, the presence of a third phase transition and the lack of amorphization up to 20 GPa by the intercalation of Ne, which we found for the high-pressure phases.

**Figure 5:**
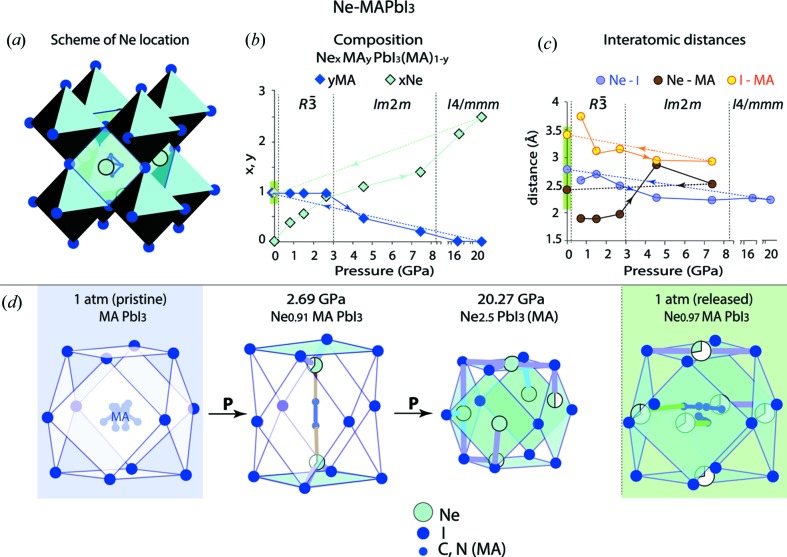
Schematic diagram of the NeMAPbI_3_ structure evolution under compression and after pressure release. Panel (*a*) indicates the arrangement of Ne atoms at the faces of the primitive perovskite cubic unit cell. These faces are represented by cyan squares formed by I atoms. Depending on pressure, some of the Ne sites can be empty or partially occupied. Panel (*b*) shows a pressure-induced change of the crystal composition. In the chemical formula, *y* and(1 − *y*) refer to MA at the long-range-ordered and the non-localized (non-periodic) positions, respectively. Panel (*c*) presents the shortest interatomic distances for Ne–I, Ne–MA and I–MA under different pressures. Panel (*d*) demonstrates the main trend of the pressure-induced structure evolution, that is of a gradual increase in the number and occupancy of Ne sites, indicated in (*a*). Unexpectedly, all these sites are also partially occupied in the released crystal.

Fig. 5 ▸ illustrates the evolution of the NeMAPbI_3_ structure under compression.

First, we observe evolution of the compound composition [Fig. 5 ▸(*b*)]: (i) a gradual pressure-induced growth in the Ne content and (ii) a gradual disappearance of the MA cation from the detectable, *i.e.* periodic over a long range, positions.

The observation (i) concerns positions of Ne atoms and their corresponding site occupancies. The intercalated Ne atoms are located in large voids of the inorganic framework, more precisely at the faces of the primitive perovskite cube [Fig. 5 ▸(*a*)], with a gradual increase in the occupancy of these sites upon compression [Fig. 5 ▸(*d*) and Figs. S4a and S5 in the supporting information]. The four neighbouring I atoms surround each Ne atom. The average occupancy of Ne sites starts at approximately one-third in the low-pressure phases and goes up with pressure, eventually approaching one when Ne atoms sit at each face. At 20.27 GPa, Ne sites are fully occupied for the faces of only four out of six possible orientations, and the remaining two are half occupied. One can therefore expect that even higher pressure is required in order to achieve the full occupancy. The corresponding composition, Ne_3_PbI_3_(MA), reflects the maximum possible Ne content in the structure.

**Figure 6:**
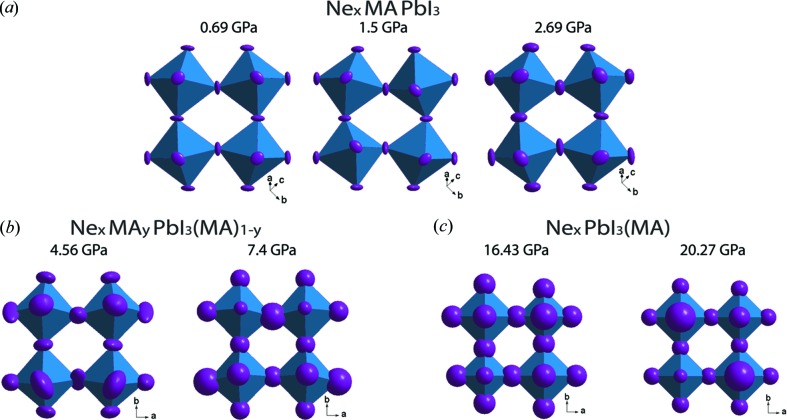
Pressure-dependent atomic displacement parameters (ADP) of I atoms for the representative fragment of the inorganic framework. Panel (*a*) shows the fragment under three pressures with the long-range-ordered MA cation. Panel (*b*) shows the fragment under two pressures with the partially long-range ordered MA cation. Panel (*c*) presents the fragment corresponding to the absence of the long-range-ordered MA cation. The ADP ellipsoids are shown with 50% probability for each pressure.

The observation (ii) needs some extended explanation. A reversible transformation, which restores all the long-range-ordered positions of MA in the released crystal at ambient pressure (Fig. 3 ▸), indicates that this cation can be in two different states in the high-pressure crystal: the long-range-ordered state (*i.e.* periodic) and the randomly distributed one (*i.e.* non-periodic). In order to emphasize these two states of MA, we use the Ne_*x*_MA_*y*_PbI_3_(MA)_1–*y*_ designation for the chemical formula, where indices *y* and (1 − *y*) denote the relative quantities of the long-range-ordered and the randomly distributed MA, respectively. The presence of two states for the MA cation is a direct confirmation of the assumption that the long-range ordering of MA is violated under high pressure, proposed by Ou *et al.* (2016 ▸), Capitani *et al.* (2016 ▸) and Szafrański & Katrusiak (2016 ▸). According to this assumption, a lack of long-range ordering of MA leads to destruction of the inorganic (Pb,I)-framework, which is kept by the I–MA periodic interactions. Indeed, a decrease in the amount for long-range-ordered MA leads to a huge increase in the atomic displacement parameters for I atoms (Fig. 6 ▸), which is a sign of the destruction of the inorganic framework. Destruction of the inorganic framework leads to compound amorphization. However, we observed that with the Ne PTM, the periodic crystal structure still exists up to 20 GPa. The reasons of this can be understood from the analysis of the Ne–I, Ne–AM and I–MA interatomic distances found for the long-range-ordered MA cation [Fig. 5 ▸(*c*)]. The short distances MA—Ne of ∼1.9 Å indicating the corresponding interaction at *P* < 3 GPa can be recognized as unstable with respect to pressure, since the content of the long-range-ordered MA rapidly reduces from 0.5 (4.56 GPa) to 0.25 (7.4 GPa) per chemical formula, and the long-range-ordered MA is no longer observed for *P* ≥ 16.3 GPa. If the expected rapid amorphization does not appear, it means that stabilization of the (Pb,I)-framework is kept by another stabilizer, which is different from MA; it is Ne atoms in the considered case. Indeed, for *P* > 4 GPa, the loss of the long-range order for more than 50% of MA switches the shortest interatomic distance (*i.e.* the strongest interaction of Ne) from Ne—MA to Ne—I [Fig. 5 ▸(*c*)]. Consequently, the Ne—I interactions can be considered as the cause of stabilization of the framework, preventing the amorphization.

The stabilizing role of Ne under high pressure is also confirmed by the distortions observed in the (Pb,I)-framework: the minimal distortions (the I—Pb—I angle ≃ 88.4–90.3° instead of the ideal 90° and the Pb—I—Pb angle ≃ 178–180° instead of the ideal 180°) were found in tetragonal structures at the highest pressure, when the long-range order MA is lost and the content of Ne is maximal (Table S3).

Thus, the progressive intercalation of Ne is the general trend in the pressure evolution of the NeMAPbI_3_. The phenomenon is driven by the Ne—I interaction, which is substantially enhanced above 4 GPa.

Surprisingly, the occupation of Ne sites in all directions is preserved in the released structure under ambient pressure, but with a much smaller probability of approximately one-third [Fig. 5 ▸(*d*)]. The mean Ne–MA and Ne–I distances in the released structure, 2.83 Å and 2.78 Å, respectively, are very similar [Fig. 5 ▸(*c*)]. The minimum Ne–MA distance of approximately 2.3 Å in the released crystal can be responsible for this stabilization. However, this is the distance between the partially occupied Ne and MA sites, so it is problematic to say with certainty whether such separation is indeed realized.

In the 0–20 GPa pressure range the bulk modulus B_0_, calculated using the Birch−Murnaghan model (Birch, 1947 ▸), varies from 5.3 GPa at the lowest to 10.6 GPa at the highest pressure. These numbers fall within the spread of values in the literature (Capitani *et al.*, 2016 ▸; Jaffe *et al.*, 2016 ▸) even for the crystalline phase at 20 GPa, which was absent for other PTMs.

### 3.2. The high-pressure ArMAPbI_3_ compounds   

The structure was determined at pressures of 0.18, 0.49, 0.98, 1.34, 2.1, and 2.39 GPa at room temperature (Table 2 ▸). Above 1.34 GPa an amorphous phase appears, coexisting with the crystalline one. The amorphous contribution grows rapidly with increasing pressure until no crystalline phase can be observed above 3.6 GPa (Fig. S2). This amorphization is irreversible in our experiments.

The tetragonal unit-cell parameters, characteristic for the ambient conditions, have been identified in the ArMAPbI_3_ structure up to 1 GPa [Table 2 ▸ and Fig. 4 ▸(*b*)(i)]. The space group *P*4_2_ *bc*, which is new for the title compound, turned out to be the best fit in the 0.18–0.98 GPa pressure range. Only one transformation of this unit cell was detected for ArMAPbI_3_, happening between 1 GPa and 1.3 GPa. The pseudo-cubic orthorhombic unit cell [Fig. 4 ▸(*b*)(i)] and the *Immm* space group are characteristic for the lattice for *P* > 1 GPa (Table 2 ▸). No distortion in the long-range order was detected for the MA cation. In comparison to NeMAPbI_3_, growth in the Ar content as a function of pressure is 5.5 times larger, reaching the composition Ar_1.4_MAPbI_3_ at 1.34 GPa. In the case of Ne PTM, the nearly equivalent composition, Ne_1.41_MA_0.25_­PbI_3_(MA)_0.75_, occurs at 7.4 GPa (Tables 1 ▸ and 2 ▸). This indicates a much stronger interaction of MAPbI_3_ with Ar than with Ne.

**Figure 7:**
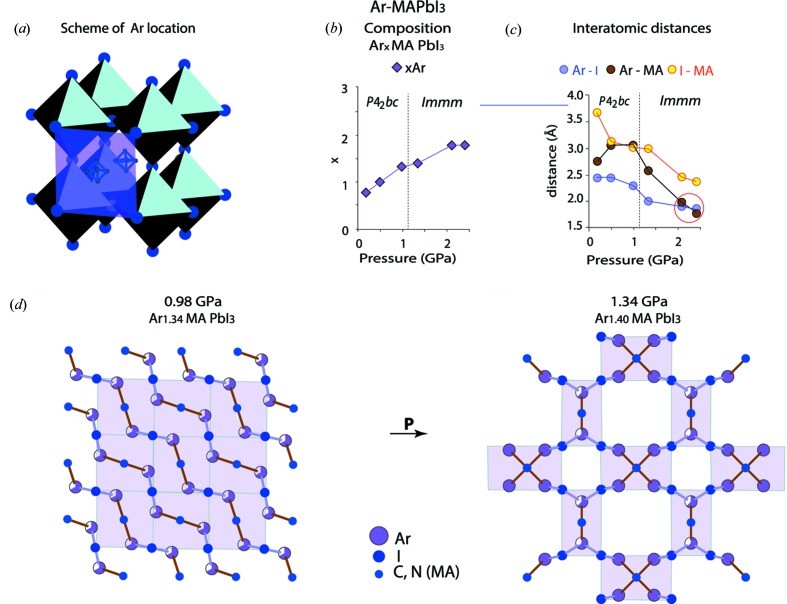
Sketch of the ArMAPbI_3_ structure evolution under compression. Panel (*a*) schematically shows a plane (pinkish grey square) where the Ar atoms are located. The plane is defined by I atoms and the centre of the MA cation. Panel (*b*) shows the pressure-induced change of the crystal composition. Panel (*c*) presents the shortest interatomic distances for Ar–I, Ar–MA and I–MA under different pressures. Panel (*d*) demonstrates the main trend of the pressure-induced structure evolution: below 1 GPa, Ar, MA and I atoms are arranged in chains, along the shortest Ar–I and Ar–MA distances, shown in (*c*); under increasing pressure, the number of the Ar positions rises, and the crossing chains form a layer. Each pinkish grey rectangle corresponds to the one shown in (*a*). Pinkish grey-coloured segments indicate the occupancies of the Ar sites.

**Figure 8:**
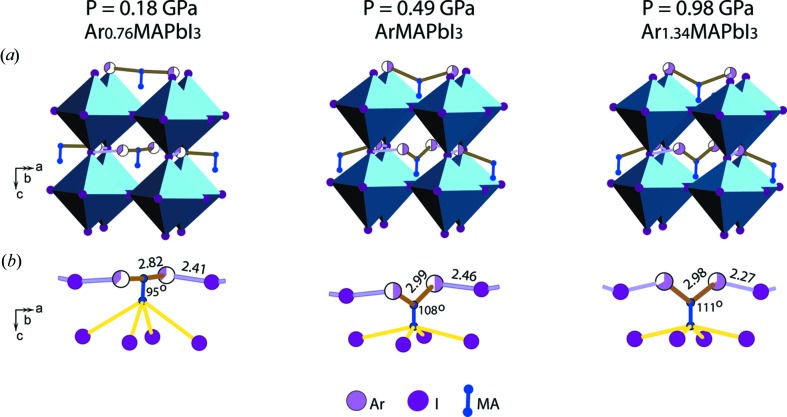
Pressure-induced transformation of the tetragonal ArMAPbI_3_ phase. (*a*) Position of the I–Ar–MA–Ar– group within the (Pb,I)-framework. (*b*) Deformation of the group configuration and its connection to I atoms. Pink-coloured segments indicate the occupancies of the Ar sites. The MA–Ar and Ar–I interatomic distances are given in Å.

The pressure-induced structural evolution of ArMAPbI_3_ (Fig. 7 ▸) is associated with a gradual growth in the Ar content [Fig. 7 ▸(*b*)], accompanied by the contraction of the Ar–I and Ar–MA distances [Fig. 7 ▸(*c*)]. For *P* < 1 GPa, the MA cations, Ar and I atoms closest to each other are arranged into chains, which polymerize in two dimensions upon further compression [Fig. 7 ▸(*d*)]. Influence of the lattice contraction on the chain geometry is illustrated in Fig. 8 ▸.

The shortest interatomic distances of ∼2 Å found for Ar–I and Ar–N (MA) at 2.1 and 2.39 GPa can be considered as a reasonable approximation if two arguments are taken into account. First, the value of 1.84 Å was theoretically predicted for the Ar–N separation for ambient pressure (Novak & Fortenberry, 2016 ▸). Second, large and prolate ADP ellipsoids of the I atoms imply an anharmonic character of the atomic displacements, which indicates that the actual Ar–I distances are longer.

Nevertheless, we consider the dramatic reduction of the minimum Ar–MA and Ar–I distances at *P* ≥ 2 GPa [circled by red line in Fig. 7 ▸(*c*)] as a trigger for the complete and irreversible structure amorphization. In fact, we recorded a crystalline state at the time of its destruction.

Thus, the pressure-induced formation of the stable I–Ar–MA–Ar– chains and their polymerization [Fig. 7 ▸(*d*)] is at the origin of the structural evolution and amorphization of ArMAPbI_3_.

Detailed characteristics of the progressive changes in the ArMAPbI_3_ structure under pressure are also given in the supporting information (Figs. S4b, S6 and S7).

### 3.3. Different impact of Ne and Ar as a pressure transmitting medium   

The present results show that both Ne and Ar interact with MAPbI_3_, but in different ways. Interactions between Ne and I atoms stabilize the inorganic (Pb,I)-framework up to 20 GPa and suppress amorphization of the crystal, despite the vanishing of the long-range ordering of MA. Ne serves as a structural stabilizer instead of MA for pressures higher than 4 GPa. Unlike Ne, Ar interacts with both I and MA even under low pressure of 0.18 GPa. These interactions accelerate the irreversible amorphization, which starts at *P* > 2 GPa. The difference between the influences of Ne and Ar is clearly related to the difference in their chemical activities, which are defined by their electronic configurations, *i.e.* their atomic radii being 0.38 Å for Ne and 0.71 Å for Ar.

The photo-luminescence (PL) microscopy images and steady-state PL spectra acquired under ambient pressure revealed that the PL properties were strongly dependent on the type of PTM and on the maximum pressure attained. Using the dual wavelength excitation technique (Mor *et al.*, 2016 ▸) these observations, described in detail in Section S6, point to a markedly higher degree of amorphization for MAPbI_3_ single crystals exposed to high pressure in Ar than in Ne. Thus, the optical characterization of MAPbI_3_ supports the conclusions of the X-ray study that the large difference in the atomic radii of Ar and Ne leads to significantly different effects of high pressure on the crystallinity of MAPbI_3_ after pressure release.

### 3.4. Influence of Ne and Ar on the methylammonium cation mobility   

The role of the mobility of the MA cation is often discussed (Motta *et al.*, 2015 ▸; Ou *et al.*, 2016 ▸; Capitani *et al.*, 2016 ▸; Szafrański & Katrusiak, 2016 ▸). The high-pressure amorphization is also directly linked to this phenomenon. According to Ou *et al.* (2016 ▸), Capitani *et al.* (2016 ▸) and Szafrański & Katrusiak (2016 ▸), the strong pressure-induced interaction between the MA cations and I atoms leads to the absence of the MA periodicity, which was considered as a template for the stability of the (Pb,I)-framework. Following this interpretation, our results support the following scenarios of the influence of Ne and Ar on the MA cation behaviour. In both cases, Ne–MA and Ar–MA interactions minimize the MA mobility by fixing the position of the MA dumbbell (the log-range-ordered form of MA). In particular, this explains the absence of cubic symmetry for the high-pressure phases.

In the case of Ne–PTM, up to ∼3 GPa, MA interacts with both Ne and I; that stabilizes its long-range ordering. For *P* > 3 GPa, intensifying interaction between MA and 12 neighbouring I atoms forces this cation to lose its long-range periodicity, first partially (for 3 GPa < *P* < 8 GPa) then completely (for *P* > 8 GPa). However, the Ne–I interaction becomes sufficiently strong to stabilize the (Pb,I)-framework. This prevents amorphization and maintains the crystalline state up to 20.27 GPa. These pressure-induced Ne–I interactions remain after decompression.

In the case of Ar PTM, MA cations interact with Ar to form –I–Ar–MA–Ar– chains as Ar enters the crystal. Formation of the chains prevents strong interaction between MA and I and, consequently, stabilizes the long-range periodicity of MA. At 1.34 GPa, some of the cations still exist outside of the chains. The uncovered cations stimulate additional absorption of Ar by the crystal, and all MA cations are involved in the polymerized chains at 2.39 GPa, but some of the Ar–MA and Ar–I distances are too short. On the one hand, it points to strong MA–Ar interactions, which benefit from the competition with the MA–I ones. On the other hand, the I–Ar–MA–Ar– chains get crowded in the restricted space between the PbI_6_ octahedra and they crush the structure. This means collapse of the structure. Despite somewhat speculative character, this scenario can explain both the small observed contribution of the crystalline phase at 2.39 GPa and the rapid irreversible amorphization upon further pressure increase.

## 4. Conclusions   

Using single-crystal synchrotron diffraction data, the crystal structure of CH_3_NH_3_PbI_3_ (MAPbI_3_) was studied under different pressures with noble gases, Ne and Ar, as pressure transmitting media creating hydro­static conditions. The following main conclusions have been drawn from the study.

(i) In the case of MAPbI_3_ compression up to 20.3 GPa, the noble gas atoms of the pressure transmitting media are not inert, but rather they form NeMAPbI_3_ and ArMAPbI_3_ high-pressure-induced compounds.

(ii) Ne mainly interacts with I atoms, preventing amorphization and stabilizing the high-pressure crystalline state up to 20.27 GPa, despite the migration of MA cations to non-periodic positions. This means a loss of the long-range ordering of MA within the crystal lattice. The high-pressure transformation is reversible and the Ne_0.97_MAPbI_3_ compound is stable at ambient conditions after decompression.

(iii) Ar interacts with both MA and I, thus forming chains and driving their pressure-induced polymerization up to *P* = 2.39 GPa. Compression of the (Pb, I)-framework destroys the polymerized structure and, consequently, the framework itself, initiating the irreversible and rapid amorphization of the compound.

(iv) The difference between the pressure-induced impacts of Ne and Ar is related to the difference in their atomic radii and, consequently, their propensity towards interatomic interactions in the restricted space between the PbI_6_ octahedra.

We believe that the findings presented can encourage the research community to conduct deeper experimental and theoretical studies of plausible chemical reactions of noble gases in the interstitial compartments of other compounds under high pressure.

## 5. Related literature   

References cited in the supporting information include: Bruce *et al.* (2011 ▸), Derenzo *et al.* (2013 ▸), Innocenzo *et al.* (2014 ▸), Klintenberg *et al.* (2003 ▸) and Wright *et al.* (2016 ▸).

## Supplementary Material

Crystal structure: contains datablock(s) global, I, II, III, IV, V, VI, VII, VIII, IX, X, XI, XII, XIII, XIV. DOI: 10.1107/S2052520619004554/dk5077sup1.cif

Structure factors: contains datablock(s) I. DOI: 10.1107/S2052520619004554/dk5077Isup2.hkl

Structure factors: contains datablock(s) I. DOI: 10.1107/S2052520619004554/dk5077IIsup3.hkl

Structure factors: contains datablock(s) I. DOI: 10.1107/S2052520619004554/dk5077IIIsup4.hkl

Structure factors: contains datablock(s) I. DOI: 10.1107/S2052520619004554/dk5077IVsup5.hkl

Structure factors: contains datablock(s) I. DOI: 10.1107/S2052520619004554/dk5077Vsup6.hkl

Structure factors: contains datablock(s) I. DOI: 10.1107/S2052520619004554/dk5077VIsup7.hkl

Structure factors: contains datablock(s) I. DOI: 10.1107/S2052520619004554/dk5077VIIsup8.hkl

Structure factors: contains datablock(s) I. DOI: 10.1107/S2052520619004554/dk5077VIIIsup9.hkl

Structure factors: contains datablock(s) I. DOI: 10.1107/S2052520619004554/dk5077IXsup10.hkl

Structure factors: contains datablock(s) I. DOI: 10.1107/S2052520619004554/dk5077Xsup11.hkl

Structure factors: contains datablock(s) I. DOI: 10.1107/S2052520619004554/dk5077XIsup12.hkl

Structure factors: contains datablock(s) I. DOI: 10.1107/S2052520619004554/dk5077XIIsup13.hkl

Structure factors: contains datablock(s) I. DOI: 10.1107/S2052520619004554/dk5077XIIIsup14.hkl

Structure factors: contains datablock(s) I. DOI: 10.1107/S2052520619004554/dk5077XIVsup15.hkl

Supporting information file. DOI: 10.1107/S2052520619004554/dk5077sup16.pdf

CCDC references: 1907811, 1907812, 1907813, 1907814, 1907815, 1907816, 1907817, 1907818, 1907819, 1907820, 1907821, 1907822, 1907823, 1907824

## Figures and Tables

**Table 1:** Crystallographic data and composition for Ne-MAPbI_3_ in the 0–20 GPa pressure range

	Ne_*x*_MAPbI_3_	Ne_*x*_MAPbI_3_	Ne_*x*_MAPbI_3_	Ne_0.97_MAPbI_3_
*P* (GPa)	0.69 → 1.5 → 2.69	4.56 → 7.4	16.43 → 20.27	1 atm (released)
Crystal system	Trigonal	Orthorhombic	Tetragonal	Tetragonal
Space group	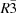	*Im*2*m*	*I*4/*mmm*	*I*422
*a* (Å)	17.3128 (9) → 17.0325 (9) → 16.7757 (9)	11.435 (3) → 11.196 (3)	10.700 (5) → 10.538 (5)	8.876 (1)
*b* (Å)	= *a*	11.437 (3) → 11.197 (3)	= *a*	= *a*
*c* (Å)	10.6019 (5) → 10.4338 (5) → 10.2267 (5)	11.442 (3) → 11.201 (3)	10.620 (10) → 10.3793 (11)	12.672 (1)
*x* in Ne_*x*_	0.42 (1) → 0.60 (1) → 0.91 (1)	1.10 (1) → 1.41 (1)	2.17 (1) → 2.50 (1)	0.97

**Table 2:** Crystallographic data and composition for Ar-containing MAPbI_3_ in the 0–2.4 GPa pressure range

	Ar_*x*_MAPbI_3_	Ar_*x*_MAPbI_3_
*P* (GPa)	0.18 → 0.49 → 0.98	1.34 → 2.10 → 2.39
Crystal system	Tetragonal	Orthorhombic
Space group	*P*4_2_ *bc*	*Immm*
*a* (Å)	8.850 (5) → 8.7468 (15) → 8.7222 (15)	12.1294 (12) → 12.0192 (10) →11.900 (11)
*b* (Å)	= *a*	12.1415 (12) → 12.0195 (10) → 11.990 (1)
*c* (Å)	12.520 (5) → 12.398 (10) → 11.9979 (15)	12.1734 (15) → 12.0213 (10) → 11.920 (1)
*x* in Ar_*x*_	0.76 (1) → 1.00 (1) → 1.34 (1)	1.40 (1) → 1.78 (1) → 1.75 (1)
