# Supplementary material for: Pressure-induced transformation of CH3NH3PbI3: the role of the noble-gas pressure transmitting media
Source: Acta Crystallogr B Struct Sci Cryst Eng Mater. 2019 May 18;75(Pt 3):361–70. doi: 10.1107/S2052520619004554 (PMC6549221; doi:10.1107/S2052520619004554)
Supplement: Supplementary file 16 [file b-75-00361-sup16.pdf]

STRUCTURAL SCIENCE
CRYSTAL ENGINEERING
MATERIALS

Volume 75 (2019)

Supporting information for article:

Pressure-induced transformation of CH3NH3PbI3: the role of the noble-gas pressure transmitting media

Alla Arakcheeva, Volodymyr Svitlyk, Eleonora Polini, Laura Henry, Dmitry Chernyshov, Andrzej Sienkiewicz, Gaétan Giriat, Anastasiia Glushkova, Marton Kollar, Bálint Náfrádi, Laszlo Forro and Endre Horváth

1 Influence of crystal quality on the HP-transformations of $MAPbI_3$

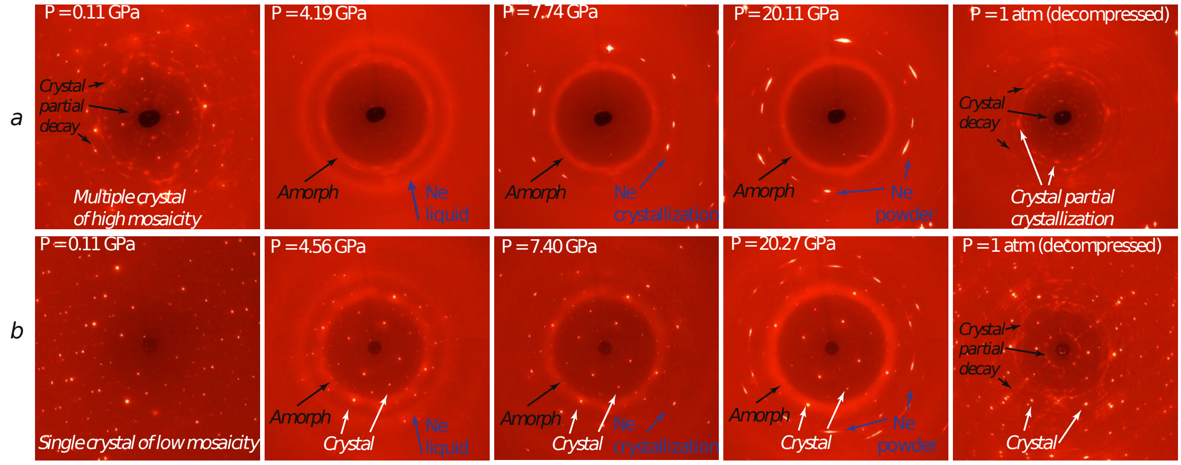

1 The high-pressure transformation of the $MAPbI_3$ crystals of different quality with Ne-PTM. (a) A multiple crystal of a high mosaicity and traces of its decay. The crystal is completely amorphous at about 4 GPa and shows only a partial recrystallization after decompression. (b) Crystal of low mosaicity and without any traces of decay. A part of the crystal keeps its crystalline state up to about 20 GPa and completely recovers its crystalline state after decompression. Two light circles observed at P = 4.19 GPa appear from two amorphous fractions: the outer one is due to Ne-liquid; the inter one is to the $MAPbI_3$ amorphous fraction. At P ≥ 7.4 GPa, only the amorphous circle is observed since Ne becomes crystalline.

2 Experimental illustrations of a pressure-dependent transformation of MAPbI3 with Ar and Ne as PTM

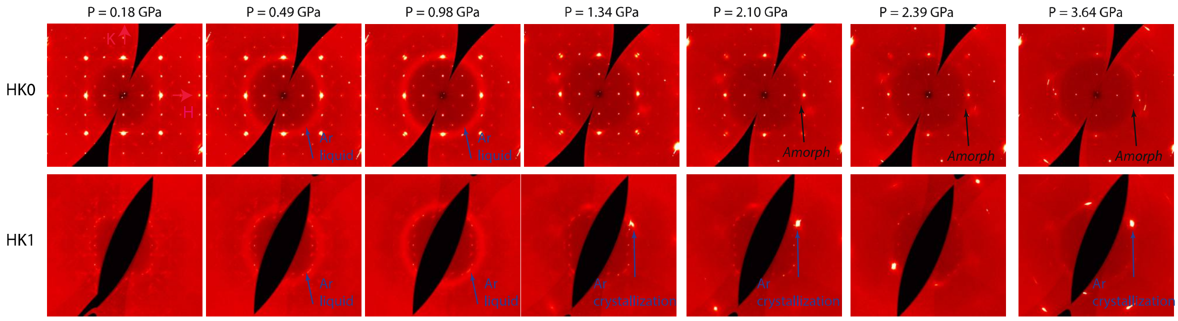

Figure S1 The high-pressure transformation of the good quality $MAPbI_3$ crystals with Ar-PTM. The reciprocal space sections HK0 and HK1 are shown. The Ar-liquid fraction gives a light circle well

visible at P = 0.49 and 0.98 GPa. This circle disappears at P ≥ 1.34 GPa due to Ar crystallization. The crystalline fraction of $MAPbI_3$ is essential at P ≤ 2.39 GPa; its presence practically disappears at P ≥ 3.64 GPa. Formation of the amorphous fraction is detectable at P ≥ 2.1 GPa (a light circle). The increase of the circle intensity indicates the increase of the amorphous fraction volume.

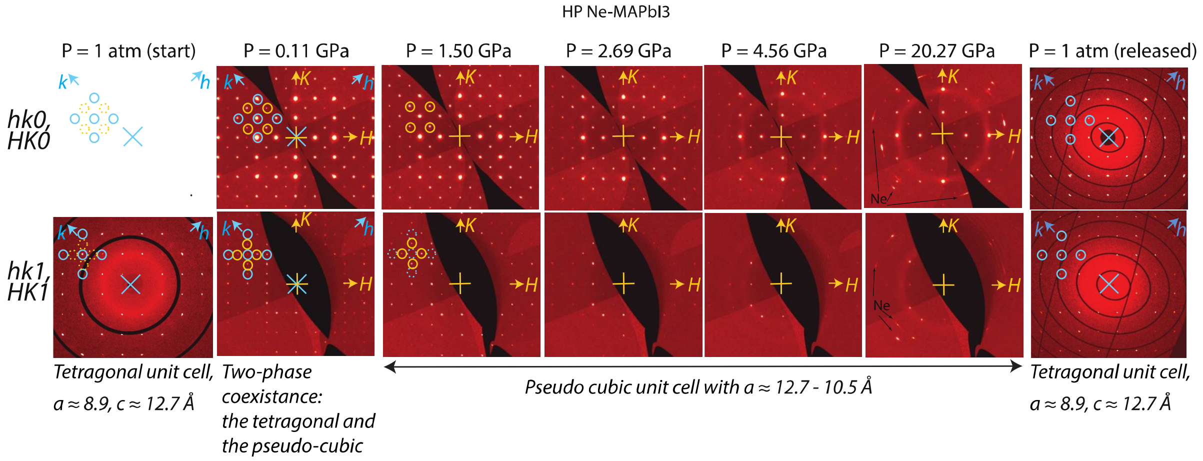

Figure S2 Details of the pressure induced phase transformations of $MAPbI_3$ with Ne as PTM. The *hk*0 (*HK*0) and *hk1* (*HK*1) planes of the reciprocal space correspond to the lattice parameters of the tetragonal (pseudo-cubic) phases. The axes *h, k* (*H, K*) and some reflections specific only for the tetragonal (pseudo-cubic) modification are underlined by blue (yellow). Superposition of the tetragonal and pseudo cubic specific reflections at P = 0.11 GPa confirms the two-phases state of the crystal. The diffraction patterns of released crystal are practically identical to those of the starting pristine crystal (space group *I*422, $a = b \approx a_{pr}\sqrt{2} \approx 8.8$ Å and $c \approx 2a_{pr} \approx 12.7$ Å).

3 Details of the crystallographic data and quality of the structure refinements

Table S1 Experimental details for Ne-$MAPbI_3$ compounds revealed at high pressure with Ne as PTM

Experiments were carried out at 293 K using Abstract diffractometer; empirical (using intensity measurements) absorption correction was applied.

	(I)	(II)	(III)	(IV)
Crystal data				
Chemical formula	$Ne_{0.42}[CH_3NH_3]PbI_3$ = $Ne_{0.42}MAPbI_3$	$Ne_{0.6}$ $[CH_3NH_3]PbI_3$ = $Ne_{0.6}MAPbI_3$	$Ne_{0.91}[CH_3NH_3]PbI_3$ = $Ne_{0.91}MAPbI_3$	$Ne_{1.10}[CH_3NH_3]_{0.5}PbI_3$ $(CH_3NH_3)_{0.5}$* =

				$Ne_{1.12}MA_{0.5}PbI_3$ ($MA_{0.5}$)*
M_r	628.5	632.1	638.3	642.55
Crystal system, space group	Trigonal**, *R*-3	Trigonal**, *R*-3	Trigonal**, *R*-3	Orthorhombic, *Im*2*m*
Pressure (GPa)	0.69	1.5	2.69	4.56
a, *b*, *c* (Å)	17.3128(9), 17.3128(9), 10.6019(9)	17.0325(9), 17.0325(9), 10.4338(5)	16.7757(9), 16.7757(9), 10.2267(5)	11.435(3), 11.437(3), 11.442(3)
α, β, γ (°)	90, 90, 120	90, 90, 120	90, 90, 120	90, 90, 90
V ($Å^3$)	2752.0(3)	2621.4(2)	2492.5(2)	1496.4(7)
Z	12	12	12	8
Radiation type	Synchrotron, λ = 0.3738 Å	Synchrotron, λ = 0.3738 Å	Synchrotron, λ = 0.3738 Å	Synchrotron, λ = 0.3738 Å
Data collection				
No. of measured, independent and observed reflections	1677, 799, 392 [$I > 3\sigma(I)$]	1449, 673, 427 [$I > 3\sigma(I)$]	703, 387, 239 [$I > 3\sigma(I)$]	1481, 783, 269 [$I > 2\sigma(I)$]
R_{int}	0.078	0.051	0.060	0.119
Range of *h*, *k*, *l*	*h* = -20→20, *k* = -14→16, *l* = -11→12	*h* = -16→12, *k* = -20→16, *l* = -9→11	*h* = -20→10, *k* = -2→19, *l* = 0→10	*h* = -6→7, *k* = -10→10, *l* = -10→10
Refinement				
$R[F^2 > 2\sigma(F^2)]$, $wR(F^2)$, *S*	0.069, 0.078, 1.69	0.049, 0.059, 1.73	0.084, 0.083, 2.27	0.058, 0.073, 0.88
No. of reflections	799	673	387	783
No. of parameters	44	40	40	59
No. of restraints	0	1	1	2
H-atom treatment	No H atoms have been included into refinement	No H atoms have been included into refinement	No H atoms have been included into refinement	H-atom parameters constrained
$\Delta\rho_{max}$, $\Delta\rho_{min}$ (e $Å^{-3}$)	1.87, -1.68	1.00, -0.89	2.31, -1.20	0.40, -0.42

	(V)	(VI)	(VII)	(VIII)
Crystal data				

Chemical formula	$Ne_{1.41}[CH_3NH_3]_{0.25}$ $PbI_3(CH_3NH_3)_{0.75}$* = $Ne_{1.41}MA_{0.25}$ PbI_3 $(MA)_{0.75}$*	$Ne_{2.17}PbI_3(CH_3NH_3)$* = $Ne_{2.17}PbI_3(MA)$*	$Ne_{2.5}PbI_3(CH_3NH_3)$* = $Ne_{2.5}PbI_3(MA)$*	$Ne_{0.97}[CH_3NH_3]PbI_3$= $Ne_{0.97}MAPbI_3$
M_r	622.9	631.6	638.4	639.3
Crystal system, space group	Orthorhombic, *Im*2*m*	Tetragonal, *I*4/*mmm*	Tetragonal, *I*4/*mmm*	Tetragonal, *I*422
Pressure (GPa)	7.4	16.43	20.27	ambient pressure
a, *b*, *c* (Å)	11.196(3), 11.197(3), 11.201(3)	10.701(5), 10.701(5), 10.622(5)	10.538(5), 10.538(5), 10.379(5)	8.876(1), 8.876(1), 12.672 1)
α, β, γ (°)	90, 90, 90	90, 90, 90	90, 90, 90	90, 90, 90
V (Å^3)	1404.2 (7)	1216.3 (8)	1152.6 (10)	998.34 (15)
Z	8	8	8	4
Radiation type	Synchrotron, λ = 0.3738 Å	Synchrotron, λ = 0.3738 Å	Synchrotron, λ = 0.3738 Å	Synchrotron, λ = 0.7153 Å
Data collection				
No. of measured, independent and observed reflections	1125, 335, 163 [$I > 2\sigma(I)$]	481, 100, 39 [$I > 2\sigma(I)$]	380, 78, 32 [$I > 2\sigma(I)$]	3819, 1453, 1096 [$I > 3\sigma(I)$]
R_{int}	0.060	0.058	0.082	0.029
Range of *h*, *k*, *l*	*h* = -5→5, *k* = -8→8, *l* = -8→8	*h* = -9→9, *k* = -9→9, *l* = -6→5	*h* = -8→8, *k* = -8→8, *l* = -5→5	*h* = -11→11, *k* = -12→12, *l* = -18→18
Refinement				
$R[F^2 > 2\sigma(F^2)]$, $wR(F^2)$, S	0.057, 0.068, 0.87	0.051, 0.057, 1.44	0.043, 0.064, 1.49	0.035, 0.049, 1.73
No. of reflections	335	100	78	1453
No. of parameters	34	18	18	76
No. of restraints	1	0	0	1
H-atom treatment	–	–	–	H-atom parameters constrained
$\Delta\rho_{max}$, $\Delta\rho_{min}$ (e Å^{-3})	0.29, -0.32	0.58, -0.60	1.06, -1.25	1.43, -0.87

* Contribution of MA = CH_3NH_3 in non-localized (non-periodic) positions. ** The hexagonal unit cell is links to the body-centred pseudo cubic one by the transformation matrix (1-10 / 01-1 /0.5 0.5 0.5).

Table S2 Experimental details for Ar-$MAPbI_3$ compounds revealed at high pressure with Ar as PTM

Experiments were carried out at 293 K using Abstract diffractometer; empirical (using intensity measurements) absorption correction was applied.

	(I)	(II)	(III)	(IV)
Crystal data				
Chemical formula	$Ar_{0.76}(CH_3NH_3)PbI_3$ = $Ar_{0.76}MAPbI_3$	$Ar(CH_3NH_3)PbI_3$ = $ArMAPbI_3$	$Ar_{1.34}(CH_3NH_3)PbI_3$ = $Ar_{1.34}MAPbI_3$	$Ar_{1.4}(CH_3NH_3)PbI_3$ = $Ar_{1.4}MAPbI_3$
M_r	650.2	659.9	673.4	675.9
Crystal system, space group	Tetragonal, *P*4$_2$*bc*	Tetragonal, *P*4$_2$*bc*	Tetragonal, *P*4$_2$*bc*	Orthorhombic, *Immm*
Pressure (GPa)	0.18	0.49	0.98	1.34
a, *b*, *c* (Å)	8.850(5), 8.850(5), 12.520(5)	8.7468(15), 8.7468(15), 12.398(1)	8.7222(15), 8.7222(15), 11.9979(15)	12.1294(12), 12.1415(12), 12.1734(15)
α, β, γ (°)	90, 90, 90	90, 90, 90	90, 90, 90	90, 90, 90
V (Å^3)	980.6 (9)	948.5 (2)	912.8 (2)	1792.8 (3)
Z	4	4	4	8
Data collection				
No. of measured, independent and observed reflections	887, 211, 95 [$I > 3\sigma(I)$]	1057, 210, 99 [$I > 3\sigma(I)$]	1260, 247, 84 [$I > 3\sigma(I)$]	772, 237, 104 [$I > 2.5\sigma(I)$]
R_{int}	0.098	0.106	0.076	0.121
Range of *h, k, l*	$h = -9\rightarrow9$, $k = -9\rightarrow9$, $l = -14\rightarrow14$	$h = -9\rightarrow9$, $k = -9\rightarrow9$, $l = -14\rightarrow14$	$h = -10\rightarrow10$, $k = -10\rightarrow10$, $l = -15\rightarrow14$	$h = -14\rightarrow15$, $k = -4\rightarrow4$, $l = -15\rightarrow14$
Refinement				
$R[F^2 > 2\sigma(F^2)]$, $wR(F^2)$, S	0.049, 0.073, 1.44	0.068, 0.086, 1.80	0.079, 0.115, 1.82	0.086, 0.108, 1.40
No. of reflections	211	210	247	237
No. of parameters	28	28	28	36
No. of restraints	1	1	1	4
$\Delta\rho_{max}$, $\Delta\rho_{min}$ (e Å^{-3})	2.62, -2.36	3.78, -3.57	2.43, -2.03	0.86, -1.10

	(V)	(VI)
Crystal data		
Chemical formula	$Ar_{1.78}(CH_3NH_3)PbI_3$ = $Ar_{1.78}MAPbI_3$	$Ar_{1.75}(CH_3NH_3)PbI_3$ = $Ar_{1.75}MAPbI_3$
M_r	692.1	689.9

Crystal system, space group	Orthorhombic, *Immm*	Orthorhombic, *Immm*
Pressure (GPa)	2.1	2.39
a, *b*, *c* (Å)	12.0192 (10), 12.0195 (10), 12.0213 (10)	11.900 (1), 11.990 (1), 11.920 (1)
α, β, γ (°)	90, 90, 90	90, 90, 90
V (Å^3)	1736.7 (3)	1700.8 (2)
Z	8	8
Data collection		
No. of measured, independent and observed reflections	799, 248, 78 [$I > 2.5\sigma(I)$]	714, 247, 46 [$I > 2.5\sigma(I)$]
R_{int}	0.116	0.124
Range of *h, k, l*	*h* = -14→14, *k* = -4→4, *l* = -14→14	*h* = -14→14, *k* = -4→4, *l* = -14→14
Refinement		
$R[F^2 > 2\sigma(F^2)]$, $wR(F^2)$, *S*	0.084, 0.124, 1.01	0.066, 0.144, 1.08
No. of reflections	248	247
No. of parameters	36	36
No. of restraints	4	4
$\Delta\rho_{max}$, $\Delta\rho_{min}$ (e Å^{-3})	2.16, -1.02	1.42, -1.32

4 Selected geometry characteristics in crystal structures of the HP transformed $MAPbI_3$

Table S3 Selected distances and angles in the PbI_3-framework of the HP transformed $MAPbI_3$ at different pressures with Ne as PMT.

Pressure, GPa	Pb - I dist., Å		Average Pb - I dist., Å	I-Pb-I angles in octahedron, deg.	Pb-I-Pb angles between octahedra, deg.
0.69	Pb1:<br>3.131(4) ×2<br>3.111(7) ×2<br>3.128(5) ×2	Pb2:<br>3.108(6) ×6	3.12	89.5(2) - 90.5(2)	157.4(1)<br>157.9(2)
1.5	Pb1:<br>3.094(3) ×2<br>3.089(6) ×2	Pb2:<br>3.092(5) ×6	3.09	89.6(1) - 90.4(1)	153.84(16) ×2

	3.091(4) ×2				
2.69	Pb1:<br>3.035(10) ×2<br>3.048(19) ×2<br>2.983(14) ×2	Pb2:<br>3.090(16) x 6	3.06	89.2(4) - 90.8(4)	153.6(4)<br>154.5(5)
4.56	Pb1:<br>3.069(18)<br>2.78(2)<br>2.844(16)<br>3.074(16)<br>2.916(12)<br>2.825(12)		3.02	72(1) - 110(1)	149(1) - 173(1)
7.4	Pb1:<br>3.135(10)<br>2.50(1)<br>3.151(15)<br>2.79(2)<br>2.81(3)<br>2.86(3)		2.87	69(1) - 110(1)	141(2) - 168(2)
16.43	Pb1:<br>2.481(17) ×4<br>2.900(15)<br>2.411(15)	Pb2:<br>2.892(17) ×4<br>2.655(3) ×2	2.68	82.6(4) - 97.4(4)	180.0(5) x 3<br>170.7(8)
20.27	Pb1:<br>2.53(2) ×4<br>2.65(2)<br>2.54(2)	Pb2:<br>2.74(2) ×4<br>2.595(3) ×2	2.62	88.4(3) - 90.3(10)	180.0(5) x 3<br>177.9(8)
1 atm<br>(released)	3.160(4) ×4<br>3.144(4)<br>3.192(4)		3.16	89.57(8) - 90.43(4)	180.0(5) x 2<br>166.6(1)
1 atm<br>(start)*	3.173(1) ×4<br>3.1727(7)<br>3.1777(7)		3.17	89.96(1) - 90.04(1)	180.0(5) x 2<br>163.59(5)

* Data were extracted from Ref. Arakcheeva et al. (2017).

Table S4 Selected distances and angles in the PbI_3-framework of the HP transformed $MAPbI_3$ at different pressures with Ar as PMT.

Pressure, GPa	Pb - I dist., Å	Average Pb - I dist., Å	I-Pb-I angles in octahedron, deg.	Pb-I-Pb angles between octahedra, deg.
0.18	Pb1:<br>3.138(14) ×2<br>3.123(14) ×2	3.13	89.4(9) - 90.6(9)	176.6(8) - 180.0(5)

	3.16(5)			
	3.10(5)			
0.49	Pb1_1:	3.12	81(1) - 99(1)	180.0(5) ×2
	3.11(5) ×4			162(2)
	3.20(8)			
	3.09(9)			
0.98	Pb1:	3.09	79(1) - 101(1)	180.0(5) ×2
	3.21(5) ×2			158(1)
	3.07(5) ×2			
	3.03(5)			
	3.97(5)			
1.34	3.049(4) ×2	3.13	81.7(10) - 98.3(10)	144(1)
	3.122(7) ×2			168(1)
	3.204(10) ×2			153(1)
2.10	3.118(9) ×2	3.10	78.5(13) - 101.5(13)	149.1(12)
	3.028(5) ×2			165.9(16)
	3.160(7) ×2			144.0(7)
2.39	3.023(5) ×2	3.09	79.3(14) - 100.7(14)	139.7(10) - 165.2(14)
	3.07(3) ×2			
	3.175(10) ×2			

5 Structure illustrations

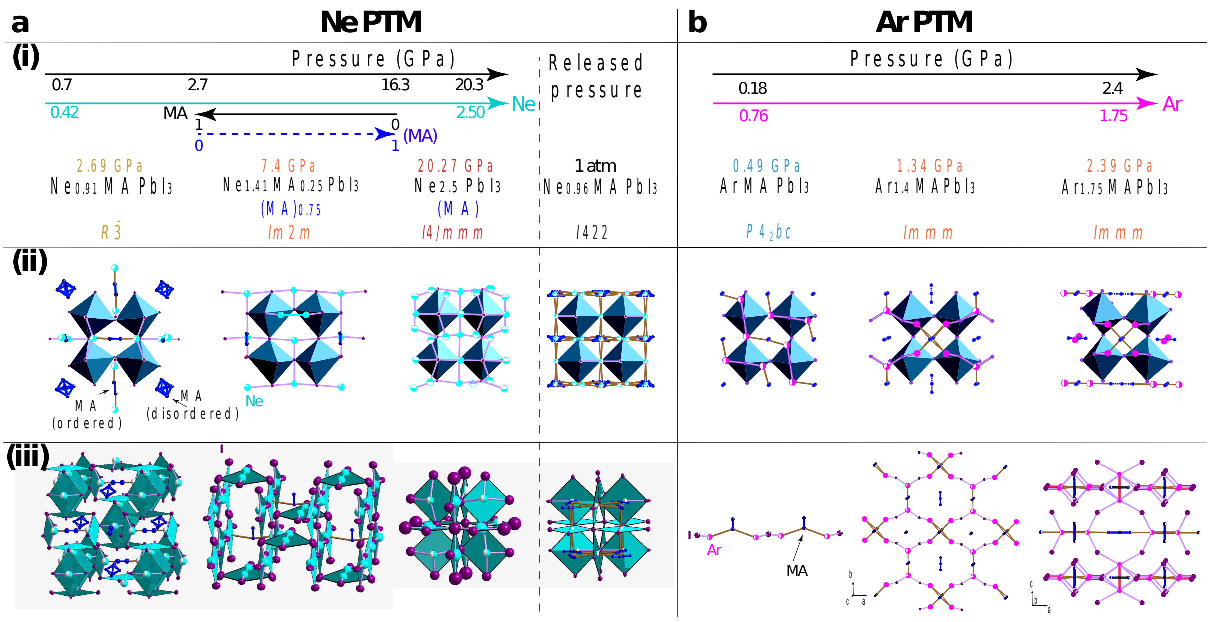

Figure S3 Typical examples of the Ne-MAPbI$_3$ and Ar-MAPbI$_3$ structures and their evolution under compression with (a) Ne and (b) Ar pressure transmitting medium (PTM). Panel (i) specifies composition and symmetry at different pressures. MA and (MA) indicate detected (long-range ordered, periodic) and undetected (non-periodic) contribution of the organic cation MA. Panel (ii) shows a representative fragment of the structure at different pressures. Panel (iii) illustrates a general

tendency of the structure evolution, which is different for Ne-$MAPbI_3$ and Ar-$MAPbI_3$. In Ne-$MAPbI_3$, Ne positions are close to the centres of the faces of a primitive perovskite cube. Increasing of pressure initiates an increase of their occupancies. Ne partially and randomly occupies all such positions in the released (decompressed) crystal at ambient pressure. In Ar-$MAPbI_3$, I-Ar-MA-Ar-chains are formed at pressure of 0.18 GPa and exist up to 1 GPa. The chains are polymerized into layers (1.34 GPa) and then into framework (2.39 GPa).

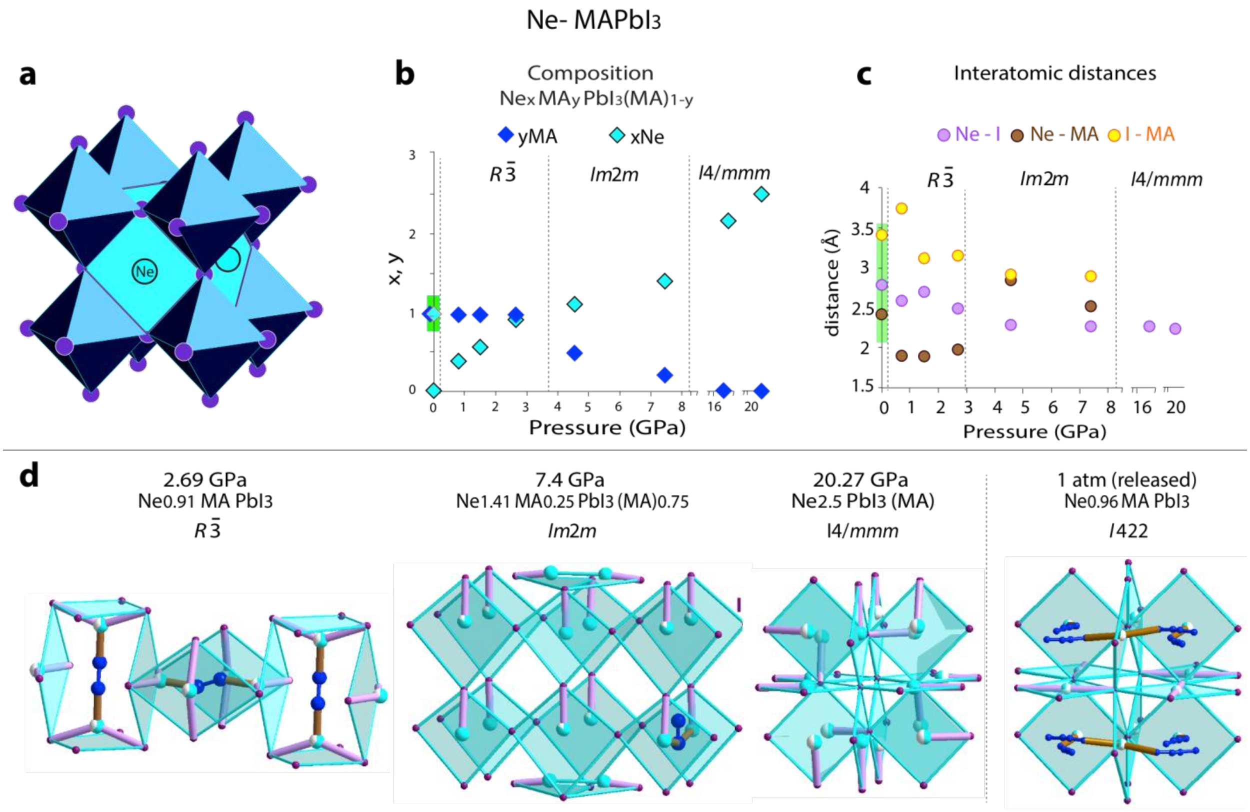

Figure S4 Evolution of the Ne-$MAPbI_3$ structures under compression and after pressure release. Panel **a** specifies a scheme of Ne positions in faces of the primitive perovskite cubic unit cell. These positions underlined by cyan squares formed by I atoms and centred by Ne. Some of the positions can be empty or partially occupied depending on pressure. Panel **b** shows a change of the crystal composition induced by pressure. In the chemical formula, y and (1-y) define the long-range ordered (detectable) MA and the randomly distributed (non-detectable), respectively. Panel **c** presents the shortest interatomic contacts under different pressures. Panel **d** demonstrates the main tendency of the pressure dependant structure evolution: the Ne positions, indicated in **a**, gradually increase in their occupancy. Only ⅓ of them are partially occupied under 2.69 GPa, and all of them are occupied under 20.27 GPa. Unexpectedly, all these positions are (partially) occupied in the unpacked crystal.

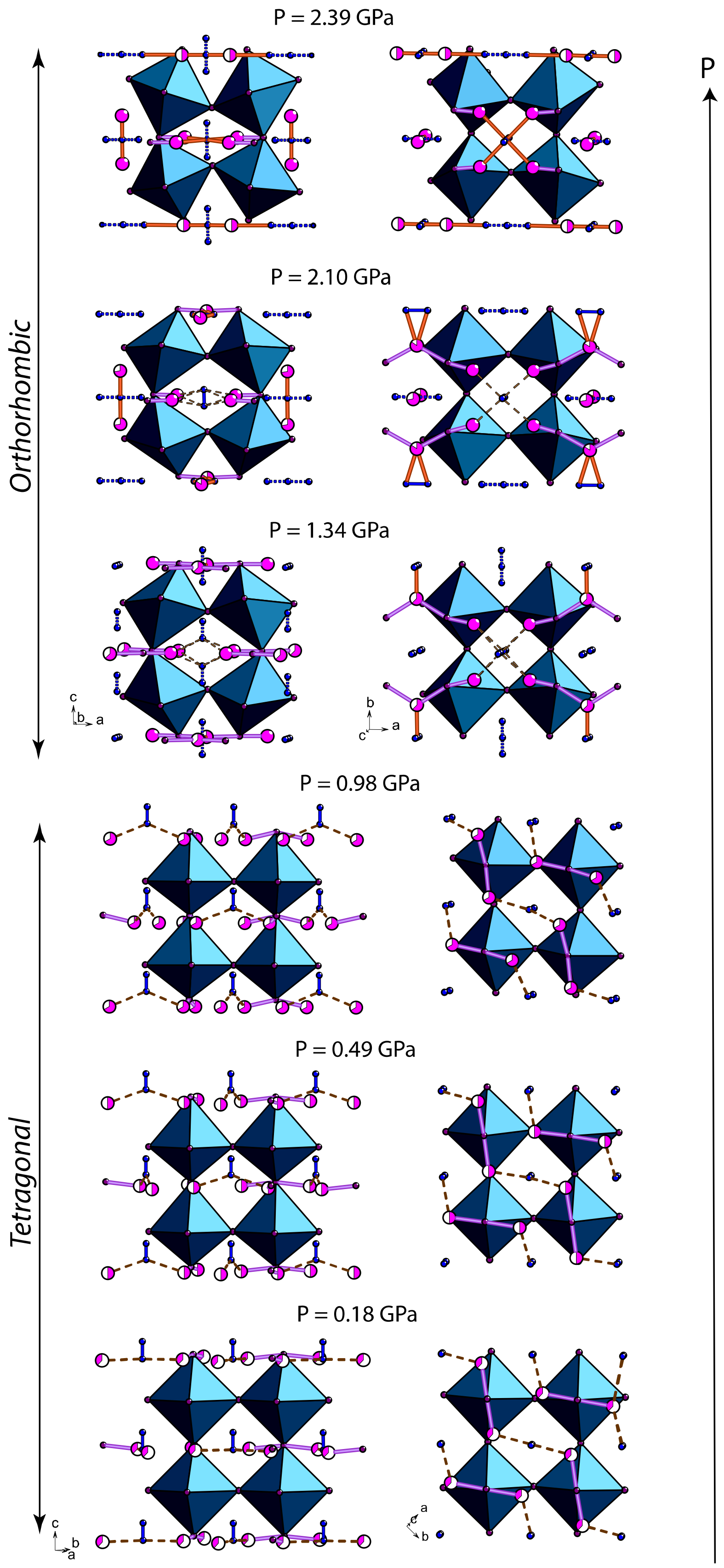

Figure S5 Evolution of the Ar-MAPbI$_3$ under increased pressure.

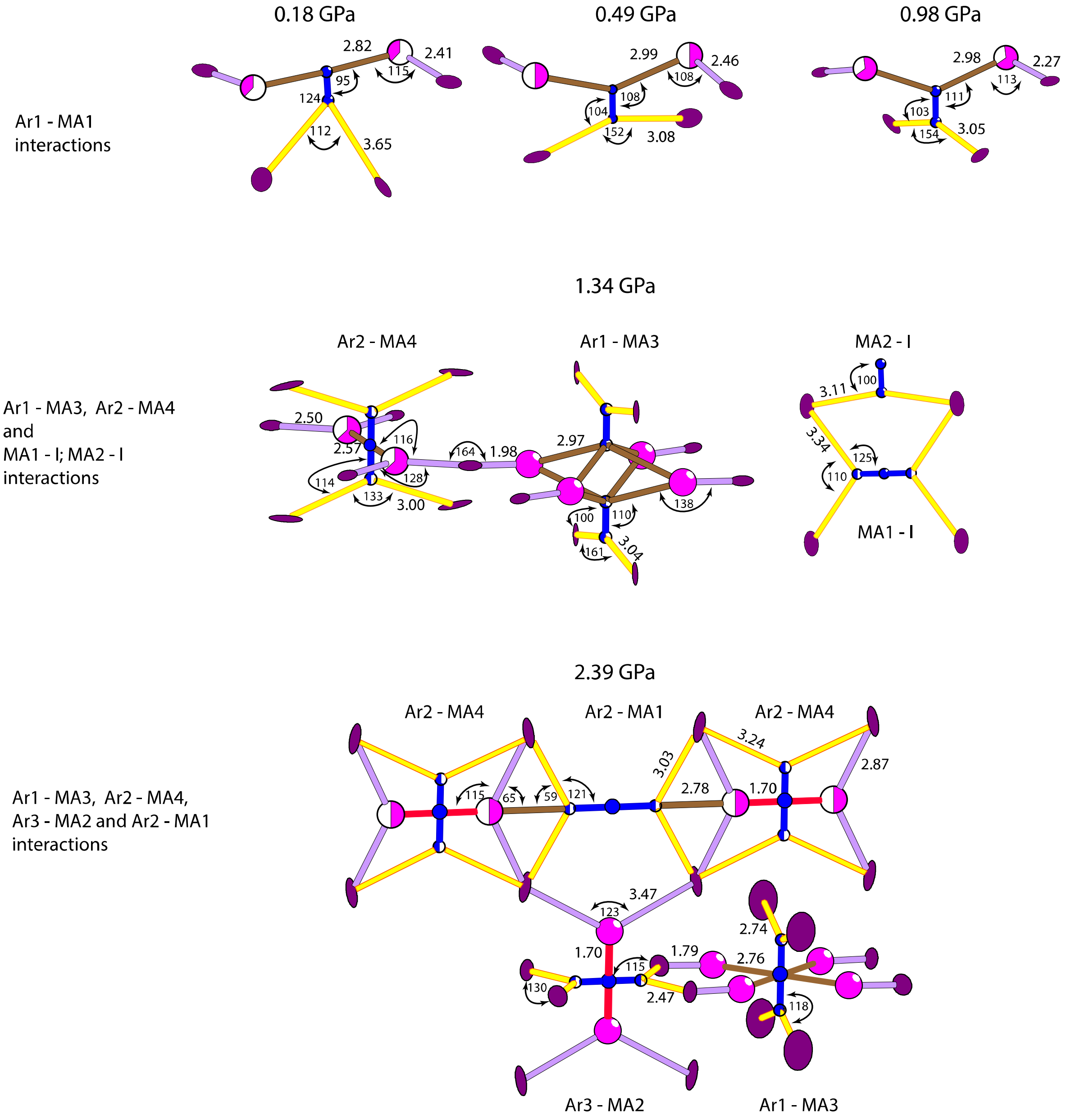

Figure S6 Pressure dependent evolution of the I-Ar-MA-Ar interactions in the Ar-MAPbI$_3$ structures. Too short Ar - MA distances (red) appeared under 2.39 GPa point to instability of the structure, which is at amorphization.

S1. Optical microscopy luminescence imaging and steady-state photo-luminescence (PL) measurements

To offer a faster and convenient detection of the amorphous phase, we performed photo-luminescence optical microscopy imaging and steady–state photo-luminescence (PL) measurements. While using two excitation wavelengths, 470 nm and 546 nm, we simultaneously acquired both luminescence microscopy images and collected the PL spectra. This novel methodological approach enabled us to

unambiguously elucidate the presence or absence of the amorphous phase in $MAPbI_3$ single crystals, which were formerly exposed to HP.

S2. Technical details

Optical microscopy luminescence imaging and steady-state photo-luminescence (PL) measurements were carried out under ambient-pressure conditions for $MAPbI_3$ single crystals that were previously pressurized with both pressure transmission media that is Ne and Ar. Similar experiments were also performed for the $MAPbI_3$ single crystals pressurized in Ne to 11.6 GPa, as well as for the intact $MAPbI_3$ single crystals (control measurements).

Specifically, the optical microscopy luminescence imaging and collection of the steady-state PL spectra were performed for $MAPbI_3$ single crystals that were formerly pressurized to 19.6 GPa and 20.27 GPa, in Ar and Ne, respectively. These studies were performed under ambient-pressure condition, *i.e.* after the pressure release and removal of the $MAPbI_3$ crystals from the DAC. Moreover, the steady-state PL spectra were also collected for the $MAPbI_3$ single crystals located in a fully assembled DAC and pressurized in Ne to 11.6 GPa. The latter measurements were then repeated under ambient-pressure conditions, *i.e.* after the pressure release and removal of the $MAPbI_3$ crystals from the DAC.

The steady-state PL spectra were collected with using a custom-designed setup combining an inverted biological epi-fluorescent microscope (TC5500, Meiji Techno, Japan) with a compact spectro-fluorometer (USB 2000+XR, Ocean Optics Inc., USA). To gather optical images of the studied $MAPbI_3$ crystals, a digital non-cooled CCD camera (Infinity 2, Lumenera Co., Ottawa, Canada) was used. Prior to perform measurements, the $MAPbI_3$ single crystals were positioned on the standard glass microscope slides (75 mm by 26 mm, 1 mm thick). A tiny amount of silicon grease was used to immobilize $MAPbI_3$ crystals on microscope slides.

In this study, the steady-state PL spectra were recorded upon illumination with two excitation wavelengths, λ_{exc}, of 470 nm and 546 nm. These two excitation wavelengths were filtered out from the emission spectrum of the microscope's Mercury vapor 100-W lamp by implementing two dedicated sets of Meiji Techno filters, *i.e.* 11001v2 Blue and 11002v2 Green, for λ_{exc} of 470 nm and 546 nm, respectively (Mor *et al*., 2016).

The control optical microscopy luminescence imaging and steady-state PL measurements were performed under ambient-pressure condition on the pristine $MAPbI_3$ single crystals originating from the same batch as the crystals exposed to high-pressure.

S3. Ambient-pressure luminescence optical microscopy and steady-state PL measurements performed on the intact $MAPbI_3$ single crystals (control measurements)

The luminescence microscopy imaging and PL measurements were performed on the control $MAPbI_3$ single crystals, which were from the same batch as those used in the high-pressure experiments and also had roughly similar volumes, being of ~4.5×10^{-5} µL. Specifically, the control photoluminescence images were acquired under illumination with two excitation wavelengths, λ_{exc}, of 470 nm and 546 nm. Moreover, in parallel to microscopic imaging, our experimental setup enabled also to simultaneously collect the corresponding steady-state photo-luminescence (PL) spectra.

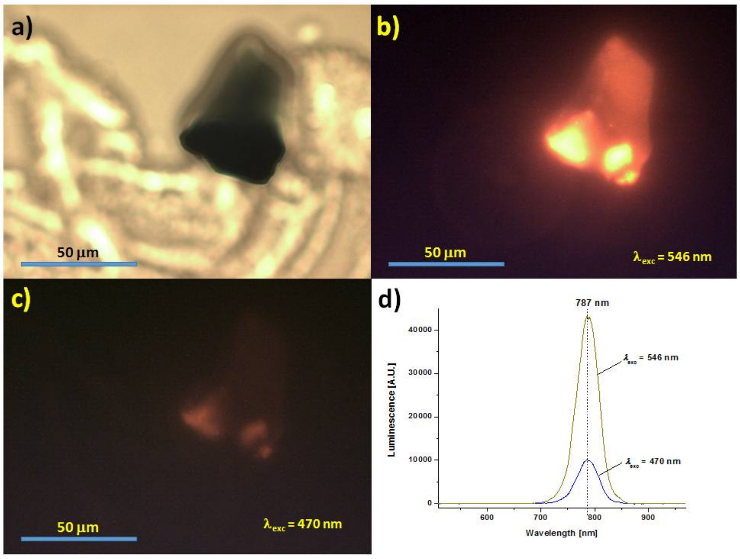

Figure S7 Typical microscopic images and steady-state PL spectra acquired under ambient-pressure conditions for a control single crystal of $MAPbI_3$: (**a**) the image obtained under visible light; (**b**) the image recorded upon excitation at λ_{exc} = 546 nm; (**c**) the image recorded upon excitation at λ_{exc} = 470 nm; and (**d**) the corresponding steady-state PL spectra obtained upon excitation at λ_{exc} = 470 nm (blue trace) and λ_{exc} = 546 nm (green trace).

The typical fluorescence microscopy images and steady-state PL spectra acquired for a control $MAPbI_3$ single crystal at two excitation wavelengths, 546 nm and 470 nm, are shown in Fig. 8.

As can be seen in Fig. 8b, upon excitation of the control crystal at 546 nm, the CCD camera picks up strong red photoluminescence corresponding to the short-wavelength wing of the characteristic photo-emission of $MAPbI_3$, which usually exhibits a luminescence peak centered at ~770 nm. As expected, this red photoluminescence, although definitely weaker, is also present in the image obtained upon excitation at 470 nm (Fig. 8c). The strongly emitting individual bright spots, which can be seen in Fig. 8b, correspond to enhanced luminescence extraction from the crystal fragments directly touching the microscope glass slide. The steady-state PL spectra, acquired simultaneously with microscopic

imaging, are shown in Fig. 8d. These spectra are depicted by the blue and green traces, for excitation wavelengths at 470 nm and 546 nm, respectively.

Taken together, the fluorescence microscopy images and PL spectra acquired for the control single crystal confirm the presence of a pure crystalline phase of $MAPbI_3$. It is also worth noting that the herein observed red-shifted emission peak of the characteristic photoluminescence of $MAPbI_3$, $\boldsymbol{\lambda}_{em\ max}$, from the typically reported value of ~770 nm (for polycrystalline $MAPbI_3$ films) to ~790 nm, is fully consistent with earlier reports suggesting the red-shifted PL peak position (along with the red-shifted optical absorption edge) for larger crystallite sizes (D'Innocenzo *et al*., 2014).

S4. Ambient-pressure luminescence optical microscopy and steady-state PL measurements of $MAPbI_3$ single crystals formerly pressurized to 19.6 GPa in Ar

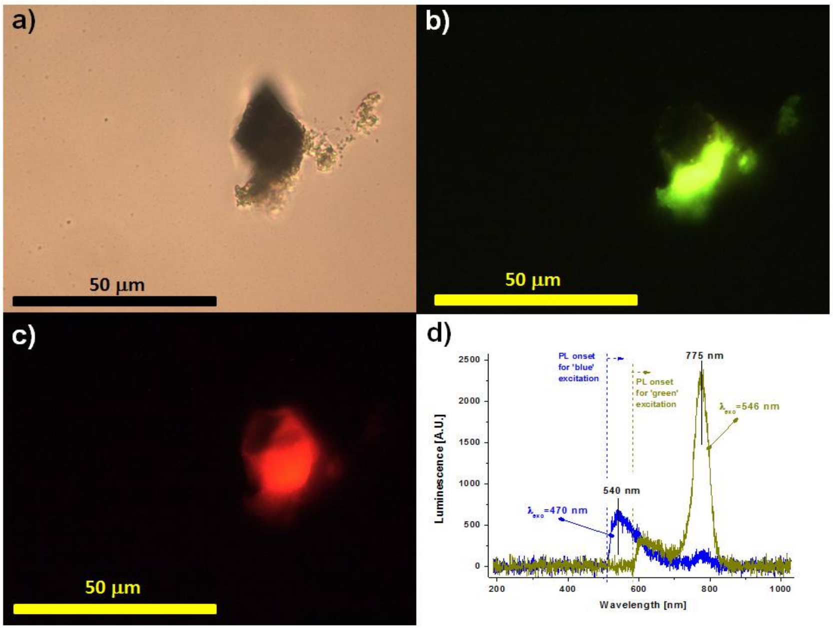

Figure S8 Microscopic images and steady-state PL spectra recorded under ambient-pressure conditions for the $MAPbI_3$ single crystal that was pressurized previously to 19.6 GPa in Ar: (a) the image obtained under visible light, (b) the image recorded upon excitation at 470 nm, and (c) the image recorded upon excitation at 546 nm; (d) the corresponding steady-state PL spectra obtained upon excitation at 470 nm (blue trace) and 546 nm (green trace). The long-pass filter-dependent onsets of PL spectra acquired under two excitation wavelengths, 470 nm and 546 nm, are indicated by the vertical broken blue and green lines, respectively.

The visible-light microscopic image recorded at ambient-pressure conditions for a single crystal of $MAPbI_3$, which was previously pressurized to 19.6 GPa in Ar, is shown in FIG. S9a. The photoluminescence images of this crystal, acquired upon excitation at 470 nm and 546 nm, are shown in Fig. S9b and Fig. S9c, respectively. As can be seen, on excitation at 470 nm, the crystal emits a strong yellow-green photo-emission (Fig. S9b), whereas on excitation at 546 nm, the CCD camera

picks up a rather weak red photo-emission (Fig. S9c). The corresponding PL spectra, acquired for this $MAPbI_3$ crystal under the excitation with both wavelengths, are shown in Fig. S9d.

The yellow-green photo-emission observed in the microscopic image acquired on excitation at 470 nm (Fig. S9b), as well as the corresponding broad PL spectrum with a stronger peak at ~540 nm and a weaker one at ~775 nm (the blue trace in Fig. S9d), point to the presence of an intermediate amorphous phase. In particular, the yellow-green emission peaking at ~540 nm can tentatively be ascribed to the rhombohedral lead iodide, PbI_2, having the optical band-gap of ~2.55 eV (Klintenberg *et al*., 2003). This broad and asymmetric PL peak is usually associated with donor-acceptor pair transitions in PbI_2 (Derenzo *et al*., 2013). The weaker photo-luminescence feature, centered at ~775 nm, can definitely be associated with the tetragonal phase of $MAPbI_3$ (Wright *et al*., 2016). As can also be seen, this PL peak gets substantially stronger on excitation at 546 nm (the green trace in Fig. S9d). We infer that a much weaker intensity of the PL peak centered at ~775 nm upon excitation at 470 nm, as compared to the excitation at 546 nm, is related to the presence of the above-mentioned amorphous phase, which filters out and attenuates the excitation wavelength (λ_{exc} = 470 nm).

Interestingly, even after prolonged illumination with both excitation wavelengths, *i.e.* λ_{exc} of 470 nm and 546 nm, we did not observed any significant alterations in the PL signal intensity. Notwithstanding, both PL signals, excited either by 470 nm or 546 nm and centered at ~770 nm, were very weak, thus pointing to a high degree of amorphization of the $MAPbI_3$ single crystal, which was formerly compressed with Ar to 19.6 GPa.

S5. Steady-state PL measurements of $MAPbI_3$ single crystals pressurized in Ne to 11.6 GPa (PL measurements performed for the crystals located in the pressurized DAC)

The steady-state PL spectra of $MAPbI_3$ single crystals pressurized with Ne to 11.6 GPa were collected through the diamond culet of a fully-assembled DAC, which was positioned on the sample stage of our inverted epi-fluorescent TC5500 microscope. A long-working-distance objective (Meiji, F10, WD=7.5 mm) was used to acquire the PL spectra from the $MAPbI_3$ single crystals located in the sample compartment of the DAC.

The actual location of two $MAPbI_3$ single crystals within the sample compartment of the DAC is shown in Fig. S10a. As can be seen, the two $MAPbI_3$ crystals were loaded along with two ruby crystals (pressure gauges). The corresponding steady-state PL spectra, acquired on excitation at two wavelengths, *i.e.* with λ_{exc} of 470 nm and 546 nm, are displayed in Fig. S10b.

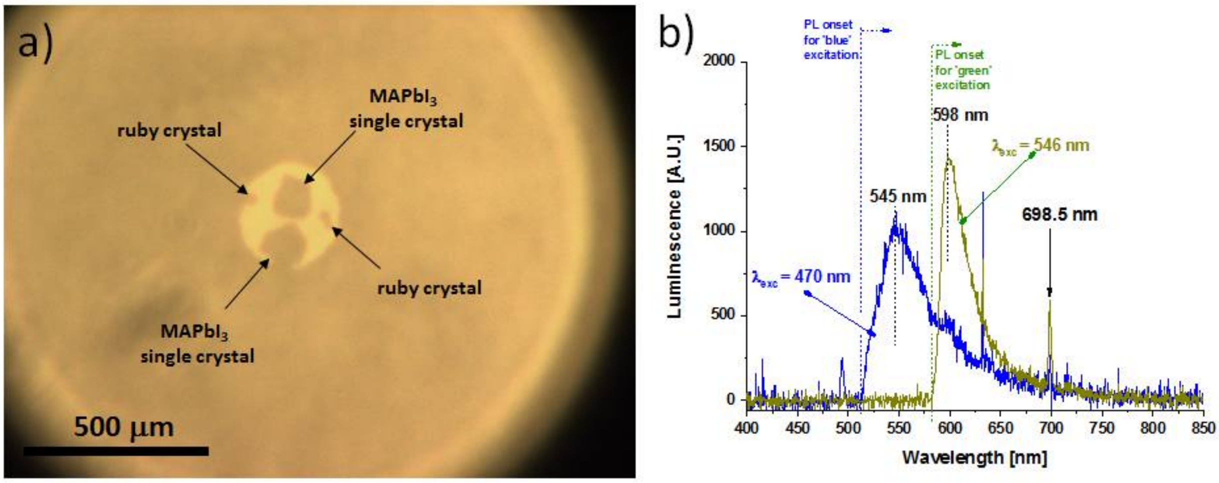

Figure S9 (a) The optical microscopy image of the sample compartment of the DAC pressurized with Ne to 11.6 GPa showing the location of two $MAPbI_3$ single crystals loaded along with two ruby crystals (pressure gauges). (b) The steady-state PL spectra collected through the diamond culet of the DAC under the optical excitation with two wavelengths, λ_{exc}, 470 nm (blue trace) and 546 nm (green trace). The sharp luminescence feature at 698.5 nm is related to the luminescence of Cr^{3+} ions in ruby crystals.

As can be seen in Fig. S10b, the characteristic photoluminescence of $MAPbI_3$ centered at ~770 nm is totally missing in both PL spectra. Instead, the only observable luminescence peak in this wavelength-range is a narrow photo-emission feature at 698.5 nm, which is related to the photo-luminescence of Cr^{3+} atoms in ruby. Being pressure-dependent, this luminescence peak of Cr^{3+} is red-shifted by 4.5 nm as reference to its position at the ambient pressure, thus confirming the high-pressure conditions (***P*** = 11.6 GPa) in the sample compartment.

The observed broadened and asymmetric photo-luminescence feature at shorter wavelengths, peaking at ~545 nm (on excitation at 470 nm), can tentatively be associated with the rhombohedral lead iodide, PbI_2, having the optical band-gap of ~2.55 eV (the blue trace in Fig. S10b) (Klintenberg *et al.*, 2003). In fact, such broad and asymmetric PL emission is usually attributed to donor-acceptor pair transitions in PbI_2 (Derenzo *et al.*, 2013). The intrinsic fluorescence of the diamond culet can also contribute to the observed photo-luminescence in this wavelength range (the blue trace in Fig. S10b), since it is well established that natural and artificially-grown diamonds reveal photo-luminescent features at 540 – 560 nm (Bruce et al., 2011).

Similarly, a slightly stronger PL signal, peaking at 598 nm under excitation with λ_{exc} = 546 nm (the green trace in Fig. S10b), can be associated with the combined contributions of the photo-luminescent response of PbI_2 and luminescent defects in diamond. The latter PL spectrum is partially cut-off on the short-wavelength side (at 590 nm) by the microscope long-pass filters.

It has to be stressed that the herein reported total suppression of the characteristic photo-luminescence of $MAPbI_3$ centered at ~770 nm for crystals pressurized in Ne to ~11.6 GPa corroborates earlier reports concerning the high-pressure induced changes in the crystalline structure and photo-luminescence properties of this metal-organic perovskite (Capitani et al., 2016).

S6. Ambient-pressure steady-state PL measurements on a single crystal of $MAPbI_3$ formerly pressurized in Ne to 11.6 GPa

After the pressure release, the steady-state PL spectra were collected at ambient-pressure conditions for the $MAPbI_3$ crystal, which formerly was pressurized to 11.6 GPa in Ne. As mentioned above, under high-pressure conditions, this crystal did not reveal any photo-luminescence. In contrast, after the pressure release, a gradual recovery of the characteristic luminescence of $MAPbI_3$, peaking at ~770 nm, was observed for this crystal. The microscopic image of the $MAPbI_3$ single crystal, which was formerly pressurized to 11.6 GPa in Ne is shown in Fig. S11.

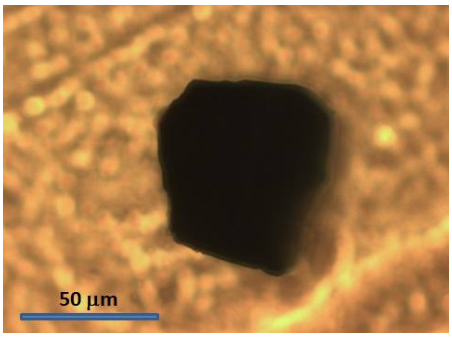

Figure S10 The visible-light ambient-pressure optical microscopy image of the $MAPbI_3$ single crystal formerly pressurized in the DAC to11.6 GPa with Ne.

The time-dependent gradual recovery of the characteristic luminescence at ~770 nm for this $MAPbI_3$ crystal at ambient-pressure conditions and upon excitation with two wavelengths, 546 nm and 470 nm, is displayed in Fig. S12.

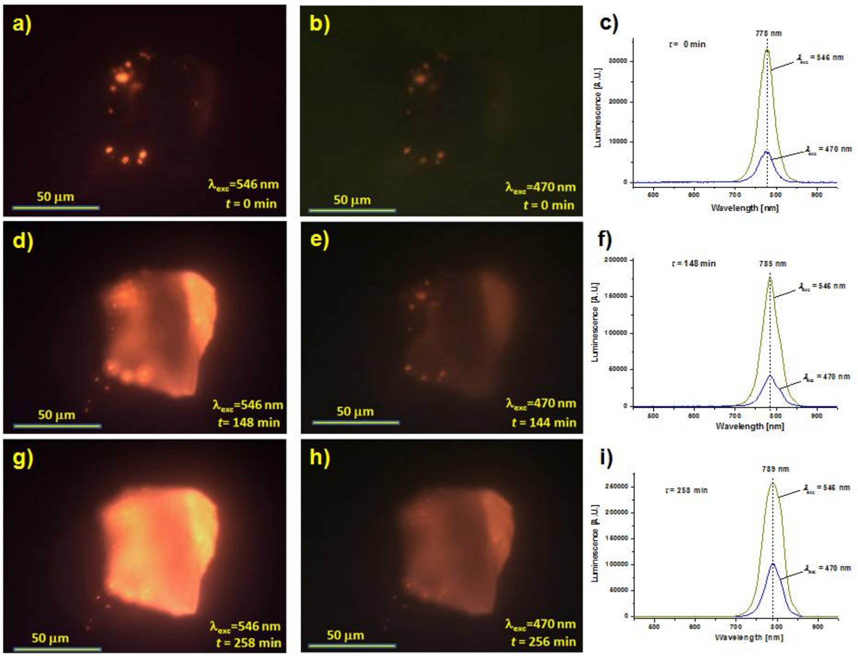

Figure S11 The time-dependent gradual recovery of the characteristic luminescence at ~770 nm for a single crystal of $MAPbI_3$ formerly pressurized to 11.6 GPa in Ne. The luminescence microscopy images recorded for the single crystal of $MAPbI_3$ on excitation at 546 nm for the progressively elongating illumination times: 0 min (**a**), 148 min (**d**) and 258 min (**g**). The luminescence microscopy images recorded for the single crystal of $MAPbI_3$ upon excitation at 470 nm for the progressively elongating illumination times: 0 min (**b**), 144 min (**e**) and 256 min (**h**). The corresponding PL spectra acquired upon excitation at 546 nm and 470 nm for the progressively elongating illumination times: 0 min (**c**), 148 min (**f**) and 258 min (**i**).

As shown in Fig. S12, upon excitation at 546 nm and 470 nm, the characteristic luminescence band of $MAPbI_3$ peaking at ~770 nm increased gradually with progressively elongating illumination times. The luminescence microscopy images shown in this figure were recorded in the beginning of the experiment (Fig. S12 a & b) and after 148 min (Fig. S12 d & e) and 258 min (Fig. S12 g & h) of illumination. It is worth mentioning that the CCD camera used in our experimental setup was only able to detect the short-wavelength portion of the NIR emission of $MAPbI_3$. In contrast, the PL spectra, acquired simultaneously (after roughly the same time-lapse intervals), provided the whole spectral information on the time-evolution of the characteristic photo-luminescence of $MAPbI_3$. These PL spectra, are shown in Fig. S12 c, f & i. As can be seen, after the subsequent illumination intervals, the marked overall growth of luminescence was also accompanied by a shift of the maximum-intensity wavelength from 778 nm (in the beginning of the experiment) to 789 nm (after 258 min of exposure to the excitation light). Similar red-shifted luminescence of $MAPbI_3$ has been reported earlier and associated with increasing crystal or grain sizes (D'Innocenzo *et al*., 2014).

Summarizing, at ambient-pressure conditions, the $MAPbI_3$ single crystal, which was formerly pressurized with Ne to 11.6 GPa, recovered the characteristic photo-luminescence emission around 770 nm, which gradually increased for longer exposure times. Therefore, the herein reported time-dependent increase of the photo-luminescence, accompanied also by the spectral red-shift, can tentatively be explained by a gradual recovery of the ordered crystalline phase of the $MAPbI_3$. It is worth noting that such behavior was not observed at ambient-pressure conditions for the $MAPbI_3$ crystal, which was formerly pressurized to 19.6 GPa in Ar. In the latter case, even after prolonged illumination, the PL intensity at 770 nm did not markedly changed and remained low. Moreover, the crystal compressed in Ar conserved a relatively strong luminescence at shorter wavelengths, which could tentatively be ascribed to the presence of an intermediate (amorphous / PbI_2-like) phase.

S7. Ambient-pressure steady-state PL measurements on a single $MAPbI_3$ crystal formerly compressed to 20.27 GPa in Ne (i.e. the $MAPbI_3$ crystal attached to a thin glass capillary)

The optical microscopy images and steady-state PL spectra were acquired at ambient pressure conditions for a single crystal of $MAPbI_3$, which was formerly pressurized with Ne to 20.27 GPa. After the pressure release and prior to PL measurements, this crystal was attached to a thin glass capillary to perform ambient-pressure XRD studies. Therefore, to avoid any further damage, the subsequent optical imaging and PL measurements were performed for this $MAPbI_3$ crystal also attached to the thin glass capillary.

The visible-light microscopic image of the $MAPbI_3$ crystal attached to a glass capillary is shown in Fig. S13.

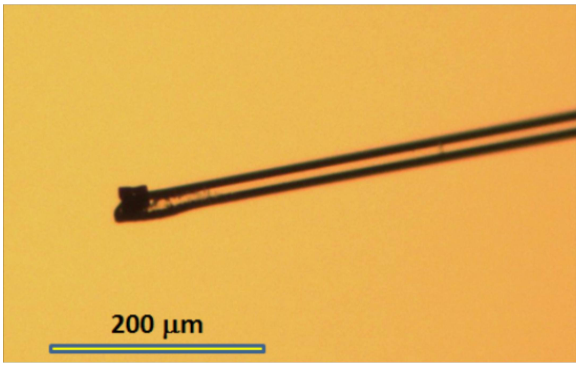

Figure S12 The visible-light optical microscopy image of the $MAPbI_3$ single crystal mounted on a thin glass capillary. Formerly, this $MAPbI_3$ single crystal was compressed in Ne to 20.27 GPa and subsequently, after the pressure release, it was used at ambient-pressure for the further characterization by XRD, optical microscopy imaging and PL measurements.

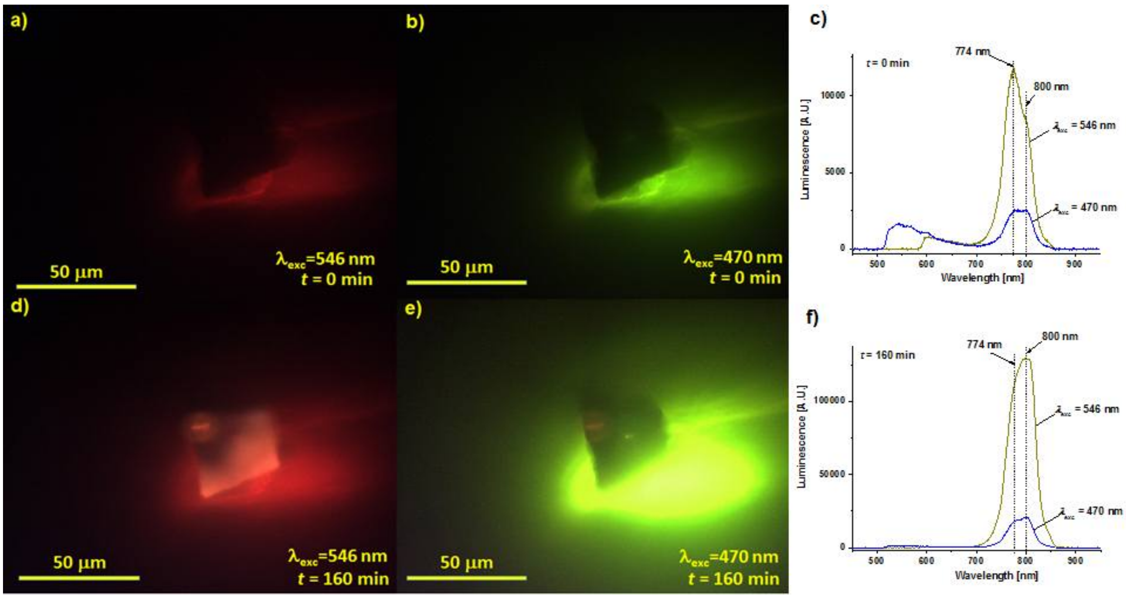

Figure S13 Ambient-pressure optical imaging and PL measurements performed for the $MAPbI_3$ crystal formerly compressed to 20.27 GPa in Ne. The luminescence microscopy images recorded on excitation at 546 nm in the beginning of the experiment (**a**) and after 160 min of illumination (**d**). The luminescence microscopy images recorded on excitation at 470 nm in the beginning of the experiment (**b**) and after 160 min of illumination (**e**). The steady-state PL spectra recorded upon excitation at 546 nm and 470 nm recorded in the beginning of the experiment (**c**) and after 160 min of illumination (**f**).

As shown in Fig. S14 a & c, in the beginning of the experiment, the characteristic luminescence of $MAPbI_3$ peaking at ~770 nm was very weak upon excitation with both 546 nm and 470 nm wavelengths. In contrast, as can be seen in Fig. S14 b & c, under the excitation with 470 nm wavelength, the yellow-green portion of the luminescence was relatively strong, thus pointing to the presence of the intermediate phase.

As it is depicted in Fig. S14 d & f, under the prolonged illumination (of 160 min), the intensity of the characteristic luminescence of $MAPbI_3$ at ~770 nm markedly increased, thus reaching ~11 times higher level than in the beginning of the experiment. Interestingly, the yellow-green portion of the luminescence, peaking at ~540 nm, increased much less, *i.e.* by ~20% only. These observations suggest that, although during the prolonged illumination the volume of the $MAPbI_3$ crystalline phase has been largely recovered, the volume of the amorphous phase (luminescent at ~ 540 nm) remained essentially constant.

As can also been seen in Fig. S14 c & f, the characteristic luminescence of $MAPbI_3$ at ~770 nm changed its spectral shape as a function of illumination time. In particular, the wavelength of the maximum emission shifted from 774 nm in the beginning of the experiment to ~800 nm after 160 min of illumination. A similar red-shift of the PL spectra has recently been reported in the context of the growing sizes of $MAPbI_3$ crystallites (D'Innocenzo et al., 2014). Therefore, the red-shift of the PL

spectra at ~770 nm observed herein can be ascribed to the progressively growing volume of the $MAPbI_3$ crystalline phase.

Overall, the ambient-pressure microscopy imaging and PL measurements performed for the $MAPbI_3$ crystal compressed in Ne to 20.27 GPa point to a much higher damage of the crystal structure than for the $MAPbI_3$ crystal, which was formerly compressed in Ne to a lower pressure, *i.e.* of 11.6 GPa. In particular, this large structural damage manifests itself by the presence of a pronounced yellow-green luminescence (peaking at ~540 nm), whereas for the $MAPbI_3$ crystal formerly compressed to 11.6 GPa its level was markedly lower. Moreover, this type of a broad emission around 540 nm was also observed for the $MAPbI_3$ single crystal formerly pressurized in Ar to 19.6 GPa and was tentatively associated with the presence of an amorphous phase. It is also worth noting that this broad emission around 540 nm was totally absent in PL spectra acquired for the intact $MAPbI_3$ crystals.

S8. Conclusions concerning the optical microscopy luminescence imaging and steady-state PL measurements

Our methodological approach, consisting of using two excitation wavelengths, *i.e.* λ_{exc} of 470 nm and 546 nm, to acquire both luminescence microscopy images and to collect steady-state PL spectra enabled us to elucidate, in a relatively simple way, the presence or absence of an intermediate (amorphous) phase in $MAPbI_3$ single crystals that were formerly exposed to high-hydrostatic pressures. Specifically, by switching excitation wavelength from 546 nm (2.27 eV) to 470 nm (2.64 eV), we could preferentially excite the amorphous fractions, containing, most presumably, the amorphous lead iodide (PbI_2) and thus having the optical band-gap of ~2.55 eV that is higher than the optical band-gap of $MAPbI_3$ (1.65 eV).

In particular, upon excitation at 470 nm, we observed a relatively strong yellow-green emission peaking at ~540 nm for a single crystal of $MAPbI_3$, which was formerly pressurized to 19.6 GPa in Ar. This broad and asymmetric PL emission, occurring at shorter wavelengths, could tentatively be associated to a high degree of amorphization and the presence of PbI_2–containing phase.

In contrast, the $MAPbI_3$ single crystals, which were formerly exposed to high-hydrostatic pressures of 11.6 GPa and 20.27 GPa in Ne, did not reveal such strong emission at shorter wavelengths, thus suggesting a definitely lower degree of the structural damage. Clearly, the acquired fluorescence microscopy images and corresponding steady-state PL spectra pointed to a higher degree of pressure-induced damage for the $MAPbI_3$ single crystal pressurized to 20.27 GPa than for 11.6 GPa. Notwithstanding, after prolonged illumination, the $MAPbI_3$ single crystals formerly exposed to high-hydrostatic pressures in Ne to 11.6 GPa and 20.27 GPa largely recovered the characteristic photo-luminescence of $MAPbI_3$ centred at ~770 nm. Interestingly, the gradual overall growth of the luminescence intensity was also accompanied by a shift of the maximum-intensity wavelength from ~775 nm to ~800 nm, in the beginning and in the end of the experiment, respectively. Such red-

shifted luminescence can be attributed to a progressive recovery (volumetric increase) of the pristine fractions of the $MAPbI_3$ crystal.

As expected, the characteristic photo-luminescence of $MAPbI_3$ centred at ~770 nm was totally suppressed for the $MAPbI_3$ single crystals pressurized in Ne to ~11.6 GPa. This latter observation is consistent with previously published data on the high-pressure behaviour of this metal-organic perovskite.

Taken together, the fluorescence microscopy images and steady-state PL spectra acquired at ambient-pressure conditions performed on the $MAPbI_3$ single crystals, formerly exposed to high-hydrostatic pressures, revealed photo-luminescence properties that were strongly dependent on the type of the pressure transmitting media (Ne or Ar) and on the maximum pressure attained. Specifically, our results point to a much high degree of amorphization of $MAPbI_3$ single crystals exposed to high-hydrostatic pressure in Ar.
